# Lifestyle-Induced Microbial Gradients: An Indian Perspective

**DOI:** 10.3389/fmicb.2019.02874

**Published:** 2019-12-17

**Authors:** Rashmi Singh, Mohammed Monzoorul Haque, Sharmila S. Mande

**Affiliations:** Bio-Sciences R&D Division, TCS Research, Tata Consultancy Services, Pune, India

**Keywords:** Indian gut microbiota, *Prevotella*, urban, tribal, bacterial diversity

## Abstract

**Introduction:** Urbanization is a globally pervasive trend. Although urban settings provide better access to infrastructure and opportunities, urban lifestyles have certain negative consequences on human health. A number of recent studies have found interesting associations between the structure of human gut microbiota and the prevalence of metabolic conditions characterizing urban populations. The present study attempts to expand the footprint of these investigations to an Indian context. The objectives include elucidating specific patterns and gradients based on resident habitat and lifestyles (i.e., tribal and urban) that characterize gut microbial communities.

**Methods:** Available 16S rRNA sequence datasets corresponding to the gut microbiota of urban and tribal populations from multiple regions of India have been rigorously compared. This analysis was carried out to understand the overall community structure, resident taxa, and their (inferred) functional components as well as their correlations with available meta-information.

**Results:** The gut microbiota of urban and tribal communities are observed to have characteristically different signatures with respect to diversity as well as taxonomic and functional composition. Primarily, the gut microbiota in tribal communities is found to harbor significantly higher species diversity and richness as compared to that in urban populations. In spite of geographical segregation and diet-related differences, gut microbial diversity was not found to differ significantly between tribal groups. Furthermore, while the taxonomic profiles of different tribal communities cluster together irrespective of their geographic location, enterotype analysis indicates that samples from urban communities form two distinct clusters. Taxonomic analysis of samples in one of these clusters reveals the presence of microbes that are common to both urban and tribal cohorts, indicating a probable transient evolutionary state. *Prevotella*, previously reported to be the dominant genus resident in Indian gut microbiota, is found to have distinct OTUs and strain-specific oligotypes characterizing resident habitats and diet patterns. Certain interesting associations between microbial abundances and specific metadata have also been observed. Overall, urban lifestyle and diet appear to impact the structure and function of gut microbial communities, and the results of this study provide further evidence of this likely detrimental association.

**Conclusion:** This study attempts to analyze, in an Indian context, the impact of urbanization on the human gut microbiota. Overall, the analysis elucidates interesting taxonomic and functional signatures characterizing the evolutionary transition in gut microbiota from tribal to urban.

## Introduction

The world is increasingly becoming urban, and globalization has resulted in a significant increase in the metropolitan sprawl of several cities. This spread has also led to rapid changes in the food habits and lifestyle choices of individuals (Collier and Venables, 2017; UN DESA, 2018). Outdoor physical activity in cities has become limited or difficult, owing to overpopulation, excessive traffic, air pollution, and limited public spaces (Tripathy et al., 2016). Globally, an absence of physical exercise and the domination of sedentary lifestyles stand as the fourth largest risk factor for mortality (Lopez and Hynes, 2006; Newtonraj et al., 2017). Altered dietary pattern is one of the consequences of urbanization (Popkin, 1999). It is well known that diet plays a major role in overall health and well-being (WHO | Diet nutrition and the prevention of chronic diseases, 2003; Satija et al., 2015).

As a country, India is a conglomeration of various ethnicities, cultures, and dietary habits. In line with global trends, Indian dietary patterns, especially in urban areas, have also experienced a drastic change. Urbanization and westernization have contributed to increased consumption of foods with limited nutritional benefit. Such foods, including ready-to-eat, factory-manufactured, packaged food items containing high levels of salt, sugar, and fat, have gradually replaced the consumption of naturally produced or unprocessed food items (Ramachandran et al., 1999). In urban areas, there is an increasing trend of increase in non-communicable diseases (NCDs) such as cardiovascular diseases, immune and metabolic diseases (including obesity and diabetes), and associated health issues. These diseases and disorders, termed urban-associated diseases (UADs), are believed to be after-effects of these dietary changes (Lopez and Hynes, 2006; Paul and Singh, 2017). In contrast, Indian tribal populations still follow ancient customary lifestyles and dietary patterns. Their diet comprises natural and forest-procured staple vegetarian food that is rich in proteins and fiber. Fermented foods and some non-vegetarian portions also comprise part of their dietary repertoire (Gupta, 1980; Shetty et al., 2013). While urban Indians have access to good livelihoods and excellent healthcare facilities with better affordability, tribal populations have poor access to healthcare and a lack of hygiene and sanitation and mostly depend on traditional medical practices, which include plant-based medicines, for the management of diseases (Uniyal et al., 2006; Kamble et al., 2010; Kapoor and Dhall, 2016). Consequently, Indian tribal populations mainly suffer from various infections and communicable and neurological diseases, which can be termed tribal-associated diseases (TADs). It is therefore evident that Indian urban and tribal populations exhibit differing levels of disease burden, which in turn is a holistic manifestation of various confounding environmental, and prevalent social-economic factors.

In addition to the role of the above-mentioned confounding factors, recent studies have indicated that the gut microbiota plays a major role in defining the overall health and well-being of an individual (Ramachandran et al., 1999; Lopez and Hynes, 2006; Cho and Blaser, 2012; Schnorr et al., 2014; Arokiasamy, 2018; Dominguez-Bello et al., 2019). A number of recent studies have also indicated the impact of the modern lifestyle and urbanization on the indigenous gut microbiota (Conlon and Bird, 2014; De Filippo et al., 2017; Barone et al., 2018). Although direct causation has not yet been attributed, the impact of urbanization on the gut microbiota has been widely associated with the onset, occurrence, and progression of several types of diseases and metabolic disorders (Obregon-Tito et al., 2015).

The structure, function, and succession pattern of gut microbiota of individuals are governed by factors such as age, sex, dietary profile, ethnicity, geographic location, disease status, etc (Conlon and Bird, 2014; Ticinesi et al., 2017). Individuals belonging to different geographical regions are therefore expected to have significant structural and functional variations in the composition of their gut microbial communities (Yatsunenko et al., 2012). These variations are now increasingly being viewed as one of the factors contributing to distinct patterns of disease burden in urban and tribal populations of India. Furthermore, studies have also indicated that, on account of heterogeneity, the Indian gut microbiota may not be similar to that in individuals from other parts of the world (Shetty et al., 2013; Dehingia et al., 2015).

Given this context, it would be interesting to study the evolution of the gut microbiota, which has likely undergone a transformation due to contemporary social and demographic changes that have happened in India in the last few decades. Apart from providing evolutionary insights with respect to the changes that have occurred in tribal and urban gut microbiota, such a comparative study is expected to throw light on the impact of immigration, modernization, and urbanization in shaping the gut microbiota (Fortenberry, 2013; Obregon-Tito et al., 2015). Furthermore, studying the gut microbiota of tribal populations (in the context of the gut microbiota of an urban cohort) provides scope for deciphering the constitutions of a “virgin” gut microbial community state. Given that gut microbial communities in tribal populations, in all likelihood, preserve the key attributes of a virgin gut microbial community state that is agnostic to exposure of modern medicines and lifestyle (Shetty et al., 2013; Clemente et al., 2015), such a comparative study (tribal vs. urban) can potentially serve as a model for identifying critical taxonomic and functional perturbations of microbial communities resulting from to changes in lifestyle (Fortenberry, 2013; Obregon-Tito et al., 2015).

In the current study, we have performed a comparative analysis of publicly available gut microbiota (16S rRNA gene) sequence data corresponding to Indian urban (UR) and tribal (TR) cohorts. The primary objective was to catalog, compare, and analyze cohort-specific taxonomic repertoire (along with the imputed functional potential) of gut microbial communities. The idea was to decipher the pattern of shift and evolution in gut microbiota composition with respect to urbanization and associated diet or lifestyle changes. A further objective was to attempt to characterize disease-associated signatures representing taxonomic and functional imbalances in the gut microbiota (in the context of urbanization and associated disease-risk susceptibility).

## Datasets Considered for Analysis

Publicly available gut microbiota datasets corresponding to Indian tribal and urban cohorts were analyzed in this study. Supplementary Table S1 provides a tabular view of studies that have analyzed the gut microbiota of Indian tribal and urban population cohorts. In order to minimize (to the extent possible) analysis artifacts and biases that may arise due to potential confounding factors (such as choice of sequencing platform, PCR primers, DNA extraction protocols, target-variable region of the 16S rRNA gene, etc, used in different studies) we selected two studies (one tribal and one urban) that had maximum similarity with respect to the above-mentioned confounding factors (Studies 1 and 2 depicted in Supplementary Table S1). Furthermore, given that the two chosen studies amplified the same variable region (V3-V4), this variable region was ideal for combining sequence data for performing OTU clustering and for maintaining uniformity in subsequent taxonomic classification as well as downstream analysis. Sequence data corresponding to these studies were downloaded from the NCBI Sequence Read Archive^1^. The data comprised amplicon sequences (encompassing the V3-V4 region of the bacterial 16S rRNA gene) corresponding to gut microbiome samples, i.e., stool samples, collected from:

• (a) A total of 80 healthy Indian “urban” individuals from Ahmedabad city. These stool samples were collected at the first time point in the basal phase, i.e., the non-intervention phase, of the study carried out by Tandon et al. (2018), and
• (b) A total of 75 healthy Indian “tribals” from four different geographical regions in India namely, Andhra, Assam, Sikkim, and Manipur. These tribal samples were from an earlier study by Dehingia et al. (2015). It should be noted that each of these geographical regions was home to multiple independent tribes.

## Results

A number of analytical, comparative, and correlation analyses were performed to address the following three questions. (i) How does the gut microbiota in the two population cohorts (belonging to different ethnicities and geographies) differ in terms of microbial community structure, taxonomic composition, and function? (ii) Is there a correlation between diet, bacterial community composition, and associated functions? (iii) Are there community-specific bacterial signatures, and what is their contribution to the overall disease burden?

### Bacterial Community Structure Differences in Tribal and Urban Gut Microbiota

Three alpha diversity metrics, namely, the Shannon (for quantifying diversity), Simpson (accounting for both richness and evenness), and Chao-1 (for assessing community richness) indices, were calculated from the taxonomic profiles corresponding to all individual samples. A statistical comparison of the metric values obtained (in the tribal and urban groups) was performed using the Wilcoxon rank-sum test with Benjamini-Hochberg (BH) *p*-value correction.

**FIGURE 1:**
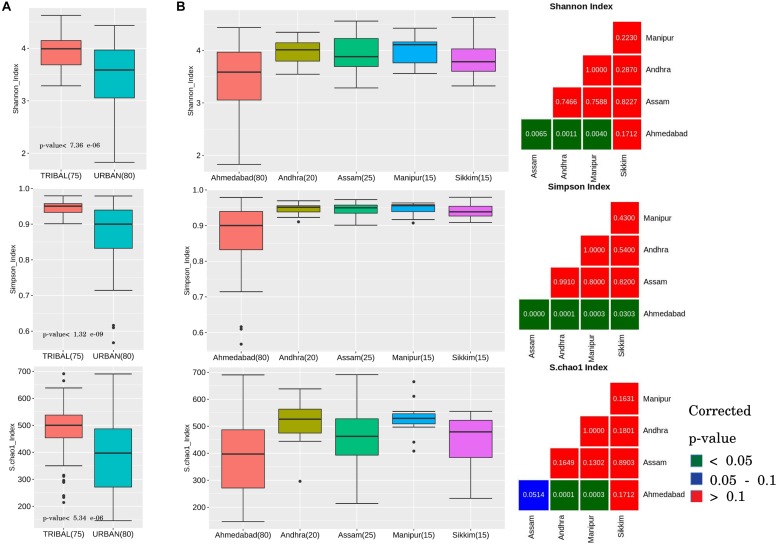
Alpha diversity trends observed in gut microbiota samples across urban and tribal samples. Changes in the gut microbial community structure were observed in terms of alpha-diversity measures: Shannon index, Simpson index, and Chao index. **(A)** Box plots depicting community diversity comparison between urban and tribal groups. Tribal samples showed significantly higher diversity (BH-corrected *p*-values < 0.1) than urban cohorts. **(B)** Box plots depicting a similar pattern of higher diversity in tribes when compared between different geographical regions (Urban, Ahmedabad; Tribal, Andhra, Assam, Manipur, and Sikkim). For each alpha-diversity measure, viz. Shannon, Simpson, and Chao, statistical comparisons with corrected *p*-values corresponding to each paired geographical region comparison are shown as a sub-plot. The colors indicated in the legend provided for the figure can be used to interpret the statistical significance of the obtained corrected *p*-value.

Comparison of the tribal and urban sample groups indicated an similar overall pattern of results for all three alpha diversity measures. The results primarily indicate that the gut microbiota of tribal communities harbored significantly higher species diversity and richness (corrected *p*-values < 0.0001) as compared to that in urban populations (Figure 1A).

Pair-wise comparisons of alpha diversity indices were subsequently performed for confirming whether the above results, observed at a cohort level (i.e., all tribal vs. all urban), also hold good if a similar comparison is performed between each of the tribal groups residing in various geographical regions and the urban cohort. In other words, statistical comparisons were performed amongst tribal groups located within different geographical regions in India (Assam, Andhra, Manipur, and Sikkim) and compared with urban samples (Ahmedabad). The results (Figure 1B) showed the Shannon and Simpson values (computed from samples from individual tribal geographical regions) to be significantly higher (except in the case of Sikkim samples) than samples from the urban region (Ahmedabad) at a corrected *p*-value of less than 0.05. Species richness (Chao-1 index) was observed to be significantly higher (corrected *p*-value < 0.001) in Andhra and Manipur gut microbiota samples in comparison to samples from the urban cohort from Ahmedabad (Figure 1B). Diets specific to individual tribal groups could be a likely reason for the observed differences in the richness index. It is interesting to note that between the tribal groups, differences in alpha diversity measures were not statistically significant (even at a corrected *p*-value < 0.1), suggesting similarity in terms of species richness amongst tribal populations.

### Differences in the Taxonomic Composition of Urban and Tribal Gut Microbiota

#### Most Abundant Genera and OTUs

The most abundant taxa (top 10 genera and OTUs) were identified based on their relative median abundances in the respective cohorts. *Prevotella copri* was found to be the dominant and most abundant species in the gut microbiota of both urban and tribal cohorts (with 61% and 36% median abundances, respectively). *Roseburia, Faecalibacterium, Alloprevotella*, and *Dialister* were among the common top five genera across both cohorts. Amongst them, while *Roseburia, Alloprevotella*, and *Dialister* were found to be more abundant in the tribal cohort, *Faecalibacterium* was observed to be relatively more abundant in the urban cohort (Supplementary Table S2).

In terms of OTU abundances, analysis of the top 10 OTUs indicated the following. While OTU_2 was observed to be relatively more abundant in the urban cohort, OTU_33 had a relatively higher abundance in the tribal cohort (Supplementary Table S2). Interestingly, a blastn search of sequences corresponding to both these OTUs against full-length 16S rRNA gene sequences sourced from the RDP database indicated best hits with the same *Prevotella copri JCM 13464* (albeit with different percent identity values). The blast identity values (listed in Supplementary Table S3 for all core OTUs in both cohorts), however, seemed to indicate a better match with *Prevotella copri JCM 13464* for OTU_33 (99.09% identity) as compared OTU_2 (96.88% identity). It is likely that the latter OTU belongs to an entirely different strain of *Prevotella copri*, sequence information for which is currently unavailable in the RDP database. Although previous studies have reported the dominance of *Prevotella copri* in Indian gut microbiota (Das et al., 2018; Tandon et al., 2018, 2019), the present study suggests the presence of community-specific strains and OTU-level differences (of genus *Prevotella*) between urban and tribal Indian gut microbiota.

#### Core Genera and OTUs

**FIGURE 2:**
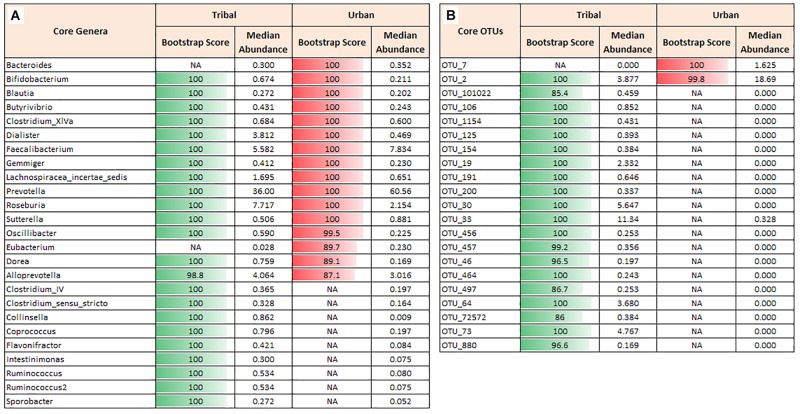
Core genera and core OTUs. Core taxa were identified for tribal and urban groups that were observed in ≥70% of the samples with a minimum abundance of 0.1% relative abundance. **(A)** Core genera in tribal and urban groups with a bootstrap score of >80. Although some genera, viz., *Prevotella* and *Faecalibacterium*, were observed as core in both communities, there is a huge difference in their median abundance between the groups. **(B)** Core OTUs in tribal and urban groups with a bootstrap score >80. Differences in the median abundance of core OTUs in tribal vs. urban communities were observed, similar to the core genera profile. A large number of OTUs that were observed to be core in the tribal community were almost absent in the urban cohort. Different OTUs for *Prevotella copri* were abundant and were found to be core in the tribal (OTU_33) vs. the urban cohort (OTU_2). NA indicates that Genus or OTU did not cross the requisite boot-strap score to qualify as a ‘core’ Genus or ‘core’ OTU, respectively.

In order to characterize the “core microbiota” across cohorts, core phyla, genera, and OTUs were ascertained using the procedure described in Ganju et al. (2016). Four bacterial phyla, namely *Firmicutes, Bacteroidetes, Proteobacteria*, and *Actinobacteria*, were identified as core in both urban and tribal populations. However, the median abundance across samples and the overall abundance distribution of the phylum *Bacteroidetes* were both found to be higher in the urban population. In contrast, the abundances of *Firmicutes, Proteobacteria*, and *Actinobacteria* were found to be higher in the tribal cohorts (Supplementary Figure S1A). At the genera level, 23 core genera across tribal samples and 16 core genera across urban cohorts were identified. Although both cohorts shared 14 core genera (Figure 2A), a significant difference was observed in their median abundances. Notably, the median values of *Prevotella, Faecalibacterium*, and *Bacteroides* were found to be higher in urban samples than in samples from tribal populations. It is interesting to note that a few genera affiliated with phylum *Firmicutes* and a genus (*Collinsella*) affiliated to the phylum *Actinobacteria* constituted the core genera that were found to be exclusive to tribal populations. Rank-normalized abundances of core genera identified in both cohorts are illustated in Supplementary Figure S1B. It is worth noting that these bacterial genera are known butyrate-producers that play a key role in maintaining good gut health in humans (Pryde et al., 2002; Louis and Flint, 2009). They are reported to be primarily involved in microbial fermentation of complex non-digestible dietary carbohydrates and host-derived glycans and the production of beneficial short-chain fatty acids (SCFAs) with anti-inflammatory properties (Esquivel-Elizondo et al., 2017).

Similar analysis performed using OTU abundance data indicated 20 and 2 core OTUs in TR and UR cohorts, respectively. OTU_2 (assigned to species *Prevotella copri*) was identified as a core OTU common to both populations (Figure 2B). However, its median abundance was found to be significantly higher in the UR population. Additionally, OTU_7 (assigned to species *Faecalibacterium prausnitzii*) was observed to be exclusively present as core in the UR population, whereas 19 OTUs constitute the core OTUs exclusive to TR populations. Details of the bacterial species and strain level affiliation of the core OTUs and their blast percentage identity values are documented in Supplementary Table S3.

#### Clustering Pattern of Gut Microbiota Samples Belonging to Urban and Tribal Communities

**FIGURE 3:**
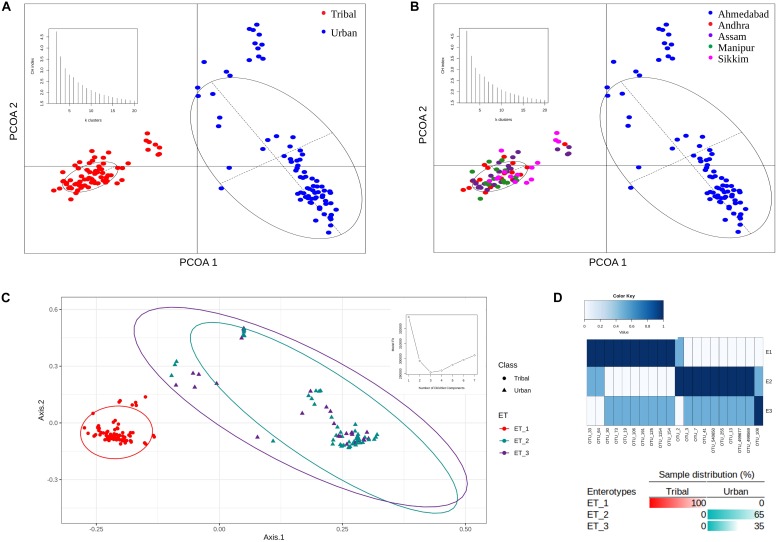
PCoA clustering of microbial abundance data based on Jensen–Shannon divergence. **(A)** Two distinct clusters were obtained for tribal and urban cohorts. **(B)** Clustering of all samples belonging to tribal and urban cohorts also revealed two distinct clusters, with all samples belonging to tribal geographical regions being clustered together and samples belonging to the urban region being segregated in a separate cluster. The inset depicts the optimal number of clusters obtained using the CH-index. **(C)** Enterotypes were identified that putatively drive the clustering pattern. The inset depicts the identification of the optimal number of enterotypes according to the model fit. **(D)** Sample distributions, with the top 20 OTU contributions to the identified enterotypes represented as an inset. Three enterotypes were observed, with ET_1 having OTUs specific to the tribal cohort and ET_2 having OTUs specific to the urban cohort, while ET_3 had a shared OTU profile of OTUs belonging to ET_1 and ET_2.

The OTU abundance profiles corresponding to the samples in both population groups were subjected to principal coordinate analysis (PCoA) using Jensen-Shannon divergence as a distance metric. The results of this ordination analysis indicated an optimal grouping of the gut microbiota samples into two distinct clusters (i.e., maximum CH-index of 2 as depicted as an inset in Figure 3A). Almost all of the samples from the four tribal geographical regions (Andhra, Assam, Sikkim, and Manipur) were observed to cluster together and were spatially separated from the samples corresponding to the urban population from Ahmedabad (Figures 3A,B).

Dirichlet multinomial models (DMM) were built using the OTU abundance data to identify specific enterotypes within the Indian urban and tribal study populations (Holmes et al., 2012). As recommended with the DMM method, the optimal number of enterotypes was ascertained by comparing model fit parameters for a range of different numbers of Dirichlet components (*k* = 1 to *k* = 7). The results indicated the presence of three different enterotypes (according to this number showing the best DMM, with the minimum Laplace score, as displayed in the inset to Figure 3C) in the OTU abundance data. The taxonomic contributions (top 20) to Dirichlet components are provided in Supplementary Table S4. Notably, while one of the enterotypes was observed to be specific to TR samples, the remaining two were found to be scattered but to be constituted exclusively of UR samples. The clustering pattern (along with the sample distribution) is illustrated in Figure 3C. The distribution of the top 20 OTUs among the three enterotypes determined is depicted in Figure 3D. The results clearly indicated that while Enterotype 1 (ET_1) comprised an exclusive set of OTUs pertaining to all TR samples, a different set of OTUs constituting Enterotype 2 (ET_2) were exclusive and highly abundant in 65% of samples from the UR population (Figure 3D). In contrast, 35% of samples from the urban population pertained to Enterotype 3 (ET_3), which had a few common sets of OTUs with both ET_1 and ET_2, with moderate abundances. This seems to suggest a mixed gut microbiota resulting from possible immigration of a subset of the population from sub-urban/tribal areas to urban areas while still retaining traces of the gut microbiota signatures that are unique to tribal populations.

#### Community-Specific Taxonomic Signatures

**FIGURE 4:**
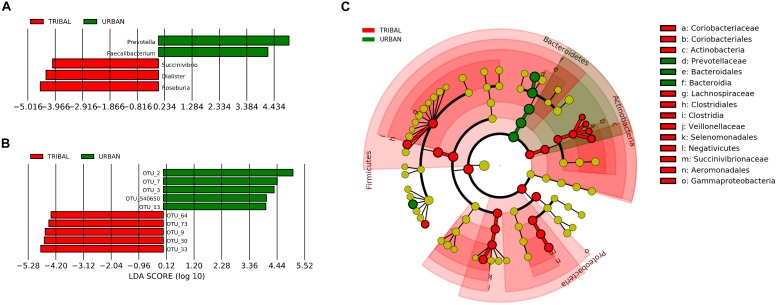
Taxa with significantly different abundance (identified using LEfSe). **(A)** OTUs with significantly different abundance in tribal and urban gut microbiota. **(B)** Genera with significantly different abundance were identified for the tribal and urban cohorts with an LDA cut-off of >4 at a *p*-value of >0.05. **(C)** Cladogram depicts identified taxa at different levels of the taxonomic hierarchy. While the phylum *Bacteroidetes* has a significantly higher abundance in the urban cohort, the abundances of taxa belonging to phyla *Actinobacteria* and *Firmicutes* were significantly higher in the tribal cohort.

A linear discriminant analysis (LDA)-based LEfSe approach (Segata et al., 2011) was employed to identify community-specific microbiota, i.e., bacterial genera whose abundance pattern in the gut microbiota was significantly different between urban and tribal populations. The results indicated five genera with a significantly different abundance (at a *p*-value cut-off of <0.05) between UR and TR samples, with an absolute LDA score of >4 (Figure 4A). Among the five genera, *Prevotella* (phylum *Bacteroidetes*) and *Faecalibacterium* (phylum *Firmicutes*) were found to have significantly higher abundances in the UR category. On the other hand, the genera *Succinivibrio* (phylum *Preoteobacteria*), *Dialister*, and *Roseburia* (phylum *Firmicutes*) were found to have significantly higher abundances in the TR category. Figure 4B shows a cladogram highlighting the lineage of features identified by LEfSe at all taxonomic levels.

To characterize differences at the most specific taxonomic level, the LEfSe approach was also employed for identifying OTUs that had significant differences in abundance between tribal and urban gut microbiota samples. The results of the analysis (Figure 4C) indicated ten OTUs with statistically significant differences in abundances (between tribal and urban samples) with an absolute LDA score > 4 at a *p*-value cut-off threshold of <0.01. These included five OTUs significantly abundant in the UR category, namely, OTU_2, OTU_3, and OTU_540650 belonging to genus *Prevotella*, and OTU_7 and OTU_13 belonging to *Faecalibacterium.* Similarly, five OTUs were found to be significantly abundant in the TR category, namely OTU_73 (affiliated to *Faecalibacterium*), OTU_64 (affiliated to *Dialister*), OTU_30 (affiliated to *Eubacterium*), OTU_9 (affiliated to *Succinivibrio*), and OTU_33 (affiliated to *Prevotella*). Overall, the taxa mentioned were observed to share a noticeable consensus with results obtained through core microbiota analysis.

The random forest (RF) classifier (Breiman, 2001) was further employed to check the efficiency of segregation between TR and UR gut microbiota taxonomic profiles (at genus as well as OTU level). For this purpose, taxonomic profiles corresponding to TR and UR gut microbiota samples were randomly split into training and test sets (described in the section “Materials and Methods”). After training and repeated cross-validation, a final classifier model was built that was subsequently validated using the test sets. The area under the ROC curve (AUC of ROC) for the trained “bagged” model attained an ideal value of 99.5% (Supplementary Figure S2A with genera as features) and 100% (Supplementary Figure S2B with OTUs as features). Assessing the efficiency of the RF classifier model with the test set samples gave a high test AUC value of 99.6% and 98.9% for genera and OTUs, respectively, indicating a high degree of segregation between gut microbiota samples belonging to the TR and UR populations.

### Differences Between Functions Inferred From Sequence Data Corresponding to Urban and Tribal Gut Microbiota Samples

The functional capabilities of the gut microbiota of urban and tribal communities were inferred using the global mapper module of the iVikodak Platform (Nagpal et al., 2019). At the outset, it should be mentioned that the functional capabilities of microbial communities (that have been analyzed, compared, interpreted, and discussed in this study) are based on algorithmic predictions that were in turn obtained using assumptions related to gene abundances, copy number of the 16S rRNA gene, quorum of genes in a pathway, etc., in various bacterial taxa. Such inferred functional profiles can be unreliable in some ecological contexts, given that they do not accurately capture and account for bacterial genomic variation within species (Zeevi et al., 2019). Furthermore, considering that gene expression is governed by a myriad of cellular mechanisms, the basic assumption employed by the current generation of function prediction algorithms (Langille et al., 2013; Aßhauer et al., 2015; Bose et al., 2015; Nagpal et al., 2016, 2019; McNally et al., 2018) based on correlation between predicted gene abundances and function may not be quite accurate. However, given the absence of metagenomic sequencing data (i.e., whole-genome sequencing data) for the two studies considered in this analysis, using such a functional inference tool was the only possible way to gain preliminary insights about the functional potential of the analyzed communities.

Most of the predicted core functions were observed to be common to the two communities. Supplementary Figure S3 depicts the abundance pattern of various core functions across all samples from both cohorts. The heatmap depicts rank-normalized functional abundances at the most specific level (KEGG pathway level 3). As a stark observation, lipopolysaccharide (LPS) biosynthesis was observed to emerge as a core pathway, with consistently higher abundance observed for a majority of the samples in the UR population in comparison to the TR population (Supplementary Figure S3). Additionally, functions such as oxidative phosphorylation (OXPHOS), citrate cycle TCA cycle (TCA), and cationic antimicrobial peptide CAMP resistance pathway were also found to be more abundant in samples from the sUR population.

Functions that showed a statistically significant difference in abundances (between urban and tribal cohorts) were identified using LEfSe (with an LDA score of >2.5 at a *p*-value cut-off of <0.001) (Supplementary Figure S4A). Additionally, the PEC (Pathway exclusion cut-off) values for identified functions (with significant differences in abundance) were determined using the ISFA module of iVikodak to ensure that the predicted pathway had a quorum of at least 80% enzymes present (Supplementary Figure S4B). The LPS biosynthesis pathway and other housekeeping pathways related to energy metabolism (OXPHOS, carbon fixation pathways in prokaryotes), nucleotide metabolism (pyrimidine metabolism), TCA, and NAFLD-related pathways were found to have statistically higher abundance in the UR cohort. In contrast, besides a couple of housekeeping pathways (aminoacyl tRNA biosynthesis and glycerophospholipid pathway), ABC transporters and propanoate metabolism pathways were observed to have statistically higher abundance in the TR cohort. In summary, although most of the predicted pathways did not appear to have a correlation with the gut microbiota structure, lifestyle, or dietary patterns pertaining to the two different communities, it was interesting to note the higher abundances of pathways such as CAMP and LPS (known hallmarks of antibiotic resistance and inflammation, respectively) in urban populations and higher abundances of beneficial functions such as propanoate metabolism in tribal populations. However, as mentioned previously, a great deal of caution would be required before drawing functional conclusions based on such predictive methods.

### Correlations Between Taxa, Predicted Functions, and Other Metadata Aspects Corresponding to Gut Microbiota in Indian Tribal and Urban Communities

**FIGURE 5:**
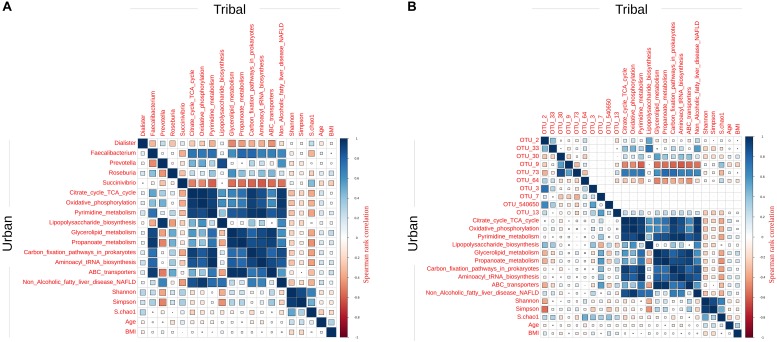
Correlation plots for Spearman rank correlations obtained using features that had a significantly different abundance in the tribal and urban cohorts. Statistically significant Spearman rank correlations obtained using **(A)** differentiating genera and **(B)** differentiating OTUs along with differentiating functions, diversity indices, age, and BMI. The upper triangle of the correlation plot demonstrates correlations in the tribal cohort while the lower triangle represents correlations in the urban cohort.

In order to investigate associations between taxa, predicted functions, and other metadata aspects of Indian tribal and urban gut microbiota, correlation analysis was performed with data corresponding to the respective communities. This analysis was performed on taxonomic profiles (and corresponding predicted functions) at both the genera and OTU levels. However, while computing correlations, only those taxa (and predicted functions) were considered that were found to have a significant difference in abundance between tribal and urban groups. Spearman rank correlations (with Benjamini-Hochberg corrected *p*-value < 0.01 above the critical *r*-value cut-off) were computed. The correlation analysis employed a rigorous bootstrap procedure (details in Methods) to minimize analytical artifacts. The identified taxa and the function pairs with significant (positive or negative) correlation between their patterns of abundance are shown in Figure 5. This figure graphically depicts the strength of correlations between genera (or OTUs) and with predicted functions, diversity indices, and metadata (e.g., age, BMI, etc.) In both panels A and B (representing the results with genera level data and OTU level data), while the upper triangle of the plot depicts correlations identified in data corresponding to the tribal gut microbiota, the lower depicts the same computed from urban data.

The results computed from genera level data (depicted in panel A of Figure 5) indicate the following main trends. The abundance of *Prevotella* in both tribal and urban populations was observed to be positively correlated (strongly) with the predicted abundances of the function lipo-polysaccharide (LPS) biosynthesis. It may be noted that the latter function is reported in the literature to be associated with low-grade inflammation (Chung et al., 2006; Cani et al., 2007; Manco et al., 2010). Furthermore, the abundance of *Prevotella* was also observed to be negatively correlated with known beneficial genera such as *Roseburia* and *Faecalibacterium* only in urban populations. Although the strength of correlations is not as high as seen with genera-level data, the correlation patterns of *Prevotella* discussed above were also observed to hold true in results computed using OTU-level data.

*Succinivibrio*, a taxon that was found to have a significantly higher abundance in tribal populations, was observed to have a strong negative correlation with several predicted functions (except LPS) in both genera as well as in OTU-level data. An inverted pattern (i.e., positive correlation) was, however, observed for the *Faecalibacterium* genus as well for its corresponding OTU, i.e., OTU_73. Interestingly, *Faecalibacterium* is reported as a beneficial gut microbe (Balamurugan et al., 2008; Sokol et al., 2008; Neish, 2009; De Palma et al., 2010; Furet et al., 2010; Rajilic-Stojanovic et al., 2011).

### Associations Between Metadata and Composition of Microbiota

In order to identify associations between the abundances of various gut microbes and confounding factors such as diet, gender, age, and BMI, etc (for which metadata information was available), suitable non-parametric statistical tests (applying appropriate *p*-value corrections for multiple testing) were employed for both “two-groups” (pooled TR vs. UR) and “multiple groups” (distinct tribal groups and UR). To enhance confidence in the results, a set of genera and differentiating OTUs were included in the analysis. Preliminarily, analysis of pooled data indicated the gut microbiota in males to be significantly more diverse (greater Shannon diversity) than that in females (Supplementary Figure S5A).

Dietary preferences were also found to be associated with the abundance patterns of a subset of genera. For instance, while samples from subjects with vegetarian diets were found to be significantly enriched with *Prevotella*, gut microbiota samples of non-vegetarian individuals were observed to harbor significantly higher proportions of *Dialister and Roseburia* (Supplementary Figure S5B). Similarly, OTUs belonging to genus *Prevotella* (OTU_2, OTU_3, abundant in the UR population) were observed to be statistically abundant in subjects following a vegetarian diet. In contrast, *Prevotella* OTU_33, OTU_9 (belonging to *Succinivibrio dextrinosolvens*), OTU_73 (*Faecalibacterium prausnitzi*), and OTU_30 (*Eubacterium rectale*) (which were more abundant in the TR population) were found to be enriched in the gut of people with non-vegetarian diets (Supplementary Figure S5C).

Associations of gut microbial taxa with age and BMI were also analyzed. For this purpose, subjects were categorized into six age groups, with a range of 5 years, increasing in each succeeding group. Similarly, based on universally acceptable BMI categorization ranges, the subjects were divided into three groups, namely, underweight, normal, and obese. Although the results obtained did not indicate statistically significant correlations between age and microbial diversity, it was interesting to note that abundances of most genera showed a declining pattern (Supplementary Figure S6A). *Prevotella*, which had an increasing abundance pattern, was, however, a striking exception to this trend. Interestingly, although the abundance of *Prevotella* was found to increase with age, OTUs belonging to genus *Prevotella* (OTU_2, OTU_3 and OTU_540650), which was enriched in the UR cohort, were found to show an increasing trend with age, while OTU_33 (belonging to the same genus), enriched in the TR cohort, was observed to have a decreasing trend with age. Similarly, while OTU_7 (enriched in UR) was observed to have an increasing trend with age, OTU_73 (enriched in TR) was observed to show a decreasing trend with age. Interestingly, both the OTUs belonged to the genus *Faecalibacterium*, for which the genus level trend with age was not found to be significant (Supplementary Figure S6B).

The results of BMI analysis indicated that the genus *Dialister* exhibited a trend of decreasing abundance with increasing BMI value (Supplementary Figure S7). At the OTU level, despite being from the same genus, *Prevotella*, OTU_2, OTU_3, and OTU_540650 were found to increase with BMI, while OTU_33 was found to have the exact opposite pattern. In addition, OTU_7, OTU_13, and OTU_73, belonging to genus *Faecalibacterium* (and with a significantly different abundance between the two populations), also showed a reverse trend wherein the abundances of OTU_7 and OTU_13 were noticed to increase with BMI, while OTU_73 followed an opposite trend (Supplementary Figure S7). Other OTUs that exhibited a decreasing pattern with BMI, namely OTU_9 (*Succinivibrio*), OTU_64 (*Dialister*), and OTU_30 (*Eubacterium*), were found to have a significantly higher abundance only in TR populations.

## Discussion

India’s urban trajectory is set to accelerate in the pursuit of faster economic growth (Collier and Venables, 2017). This transition toward urbanization has been alleged to have profound detrimental effects with respect to overall health and well-being (Saravanan et al., 2016). Urbanization has resulted in a major shift in diet and lifestyle, both of which have been shown to affect the structure and function of the human gut microbiota (Voreades et al., 2014; Singh et al., 2017). Characterizing the disparate patterns of the gut microbiota composition as well as its functions in populations living ancestral lifestyles and relatively urban lifestyles offers an opportunity to understand possible changes in microbiota with urbanization (Moeller, 2017). Recent studies have attempted to delineate the association of human microbiota in the context of ethnic differences (Fortenberry, 2013; Schnorr et al., 2014; Dehingia et al., 2015). However, little is known in the context of community-specific differences in Indian gut microbiota and their association with changes in lifestyle (Shetty et al., 2013).

In this study, we have compared the gut microbiota of tribal (with *n* = 75) and urban populations (with *n* = 80) in India. Although both studies providing the chosen data had obtained and processed the samples using more or less similar protocols (for example, sample collection, microbial DNA extraction, sequencing, and analysis, etc.), there were a few minor differences between their protocols. For instance, while the QIAGEN DNA Stool Mini-Kit was used for DNA extraction in the study of tribal populations (Dehingia et al., 2015), the Qiagen DNeasy Blood & Tissue Kit was used in the study of urban populations (Tandon et al., 2018). Between these studies, there were also minor differences with respect to the kind of assays that were used for quantification of DNA extracted from the samples. Given that NGS-based microbiota analyses are known to be extremely sensitive to experimental conditions and associated variations, the mentioned differences in protocols, although minor, could possibly have some impact on at least some of the results obtained in this study and the conclusions drawn therein.

Overall, the results of our analysis indicate intriguing correlations and trends in gut microbial structure and function with changes in community, lifestyle, and dietary patterns. The results from the enterotype analyses indicate that urban gut microbiota are driven by specific bacterial groups (OTUs). The observed overall higher diversity in the tribal cohort (as compared to the urban) in terms of gut microbiota composition can be attributed to diverse dietary patterns in these communities. However, no significant differences in diversity measures were observed between the tribal groups from distinct geographical regions, demonstrating a homogenous taxonomic gut microbiota structure among tribal populations (Figures 1, 3). It has been reported that bacterial depauperation (substantial loss of gut bacterial diversity) began in humanity’s ancient evolutionary past, and this process has significantly accelerated in recent years with the advent of modern lifestyles. Studies have suggested that humans living in industrialized societies possess the lowest gut bacterial diversity (Moeller, 2017). Some missing gut bacteria may still exist in certain communities (with primitive lifestyles and culture) or might have become globally extinct and unrecoverable (Segata, 2015). More importantly, lifestyle- or urbanization-induced depauperated microbiota may predispose human populations to certain diseases such as an increased risk of infections, autoimmune disorders, and metabolic syndromes (Marchesi et al., 2016).

The observed higher abundance of taxa belonging to *Bacteroidetes* in the urban cohort (Figure 4) supports the findings from an earlier reported study (Ou et al., 2013). This study indicated an association between the abundance of *Bacteroidetes* and a western-type diet (typically high in protein and fat), which is, in turn, known to be associated with various human metabolic diseases. It has to be noted that members of *Bacteroidetes* are known to mostly inhabit the distal gut, where they participate in provisioning the host with energy harvested from the diet through the fermentation of complex plant polysaccharides as well as otherwise indigestible polysaccharides (Singh et al., 2017). In contrast to higher *Bacteroidetes* in the urban cohort, the abundance of taxonomic groups belonging to the phylum *Firmicutes* is observed to be higher in samples from tribal communities. Most of the abundant genera (*Roseburia, Eubacterium*, and *Faecalibacterium*, etc.) and OTUs belonging to this phylum have been reported to produce beneficial metabolites like SCFA that are known to help in maintaining colon health and the integrity of gut lumen. Thus, the present results support the speculation that the tribal populations have better gut integrity than urban cohorts due to a higher abundance of *Firmicutes* (Pryde et al., 2002; Louis and Flint, 2009).

Although *Prevotella* is observed to be dominant in both the urban and tribal cohorts and its abundance patterns are observed to be in line with previous Indian gut microbiota studies (Dubey et al., 2018; Dhakan et al., 2019), its abundance is higher in the urban cohort than in the tribal group (Figure 2 and Supplementary Figure S1). Interestingly, our analyses also indicate distinct OTUs belonging to *Prevotella copri* to be dominant in the respective cohorts (Figure 4A). *Faecalibacterium prausnitzi* shows a similar pattern, wherein different OTUs of this species are found to have a significantly different abundance pattern in the two communities. The present study shows members belonging to the phylum *Bacteroidetes* to be higher in the urban group, while those belonging to *Firmicutes* are more abundant in the tribal cohorts. Since taxa belonging to these two phyla monopolize different functional niches in the human gut ecosystem, the observed differences indicate their probable functional role in disease susceptibility. It is interesting to note that a comparison of BMIs (sourced from metadata corresponding to the tribal and urban populations analyzed in this study) indicates significantly higher BMI (Wilcoxon rank-sum test, *p*-value < 0.00001) in urban individuals (Supplementary Figure S8).

We further performed functional inference analysis in order to obtain preliminary insights regarding the functional contributions of these bacterial groups and to understand which predicted functions are enriched in each community. While the abundance of predicted functions such as LPS biosynthesis is found to be significantly higher in the urban population, the predicted abundance of propanoate metabolism is observed to be significantly higher in the tribal population (Figure 5). It is interesting to note that the LPS biosynthesis pathway is known to be a marker for low-grade systemic inflammation in the human body as a result of dysbiosis and compromised gut-barrier function (Fortenberry, 2013; Hersoug et al., 2016). In contrast, propanoate metabolism is known to be a pathway that is beneficial for the host (Pryde et al., 2002; Anand et al., 2016). Propanoate has also been reported to have anti-inflammatory potential and is known to have anti-lipogenic and cholesterol-lowering effects (Lin et al., 2012). Although the marked differences in the predicted abundance pattern of these pathways in the gut microbiota of tribal and urban populations are quite intriguing, it should be noted that inferring functional capabilities from mere taxa abundances, with the assumption that they are reasonably correlated, is fraught with risk. A great deal of caution would be required before making functional conclusions based on such predictive methods applied to amplicon sequence data. The above results (pertaining to predicted functions) are therefore only indicative in nature. Follow-up shotgun sequencing experiments will need to be carried out to improve confidence about the accuracy and certainty of the functional capabilities in these environmental niches.

The findings from the present study appear to indicate that increased or decreased prevalence of certain taxonomic groups in an environmental niche might contribute to the specific-disease burden in that particular niche. In comparison with tribal communities, the incidence of NCDs is known to be higher in urban populations. Although, in this study, the urban cohort was comprised of “healthy” study participants, it is interesting to note the higher proportions of *P. copri* in urban gut microbiota samples. Although a few recent studies have indicated the positive association of *P. copri* with human disease conditions (Larsen, 2017), contrasting reports also exist indicating their selective beneficial or detrimental roles based on dietary habits (De Filippis et al., 2019).

The increasing incidence of UADs has been hypothesized to be driven by the loss of microbes essential for human health. These hypotheses assert that, due to recent changes such as toward a sedentary lifestyle, overcooking (Pérez-Burillo et al., 2018), hygiene obsession (Hunter, 2012), high antibiotic usage, and other anthropological activities for economic development, there has been a partial loss or complete extinction of some of the beneficial microbes in the UR gut micro-environment (Conlon and Bird, 2014). The results of this study seem to be in line with these hypotheses. The gut microbiota in urban communities is observed to harbor significantly lower species diversity as compared to that in tribal populations. Given these findings, it will be interesting to further evaluate lifestyle-related perturbations in microbiota in a much larger population cohort. The findings of such a study would find applicability in deciphering novel microbiome-based signatures that can quantify the level of disease burden in selected population cohorts.

## Conclusion

Diet and lifestyle changes that accompany urbanization are known to negatively impact human health. In contrast, the traditional lifestyles and diet followed by tribal communities have been shown to provide considerable health benefits. Several recent studies performed with urban study participants have also shown an association between various human diseases and changes in the composition of human gut microbial communities. Given this context, in this study, we set out to perform a meta-analysis to compare the composition of gut microbiota in urban and tribal populations in India. Gut microbiota sequence datasets corresponding to these populations were meticulously chosen so that they had a minimum number of confounding factors that could impact the results of the analysis. The results obtained seem to clearly reinforce findings from previous global studies that indicate that urban gut microbiota have significantly lower microbial diversity as compared to those of tribal populations. A number of taxa with known beneficial roles are seen to have reduced prevalence and abundance in gut microbiota samples from urban populations. Ordination analysis indicates that a subsection of the urban cohort harbor a gut microbial composition that indicates a transient evolutionary state between tribal and urban. The functions inferred from gut microbial taxonomic profiles of urban and tribal populations further seem to indicate a preponderance of specific functions and pathways in the urban cohort. These predicted functions have been previously reported to be associated with gut inflammation – a dysbiotic condition that precedes several urban-associated metabolic disorders. However, given the predicted nature of these results, further studies will be required to confirm these findings. The results also indicate interesting associations of certain commensal bacterial taxa with metadata features like age, BMI, and dietary preferences. Overall, our study shows distinct differences between the gut microbiota structure and composition of Indian urban and tribal populations, and the results indicate interesting preliminary associations with the level of disease burden prevalent in the respective populations.

## Materials and Methods

### Pre-processing, OTU Clustering, and Taxonomic Profiling

Raw sequence data were initially subjected to pre-processing using Prinseq-lite (Schmieder and Edwards, 2011) to filter out low-quality sequences (with mean phred score < 25) and sequences with insufficient length (less than 100 bp). Quality-filtered reads were subsequently processed using the VSEARCH (v2.8.0) pipeline (Rognes et al., 2016).

Primarily, a de-replication step was employed across samples, and singletons were removed, followed by pre-clustering into operational taxonomic units (OTUs) at 98% similarity with a *de novo* approach. Chimera detection and removal was subsequently performed (using UCHIME, integrated into VSEARCH) for *de novo* chimera detection (using the *uchime_denovo* command) followed by reference-based detection (using the *uchime_ref* command) with the Gold database (downloaded from http://drive5.com/uchime/uchime_download.html). The resultant chimera-free sequence data were finally clustered at 97%, and a raw OTU abundance matrix was then created. Further, to denoise the data, sparse OTUs containing <0.002% of the total number of unique high-quality sequenced reads were removed. A total of 3,296 OTUs were finally retained for further downstream analysis (Supplementary Table S5). Raw OTU abundances were rarefied for minimum number of reads using the RAM package (version 1.2.1.7) in R^2^ to eliminate biases potentially arising due to differences in the sequencing depth of various samples. The size of the sample with the lowest sequencing depth (10,678 reads) was chosen as the uniform threshold depth for rarefaction of all other samples considered in the analysis.

Taxonomic assignment of representative OTU sequences was performed using the naive Bayesian classifier implemented in Ribosomal Database Project version 2.12 (Wang et al., 2007). OTU-level abundances were also appropriately cumulated at higher taxonomic levels (e.g., phylum, family, and genus, etc.) for downstream analyses (Supplementary Table S6). Furthermore, to obtain species-level information, pair-wise alignment of representative OTU sequences was carried out using blastn against the 16S Microbial database (downloaded from ftp://ftp.ncbi.nlm.nih.gov/blast/db/16SMicrobial.tar.gz) with >90% identity, at an *e*-value cut-off of 1e-5.

### Estimation of Community Diversity

Alpha diversity indices for individual samples were computed using the R-Vegan (v2.5-3) package^3^. Three diversity indices, viz., the Shannon, Simpson, and Chao indices, were computed for each of the samples. Subsequently, a Wilcoxon rank-sum test and FDR correction [Benjamini Hochberg (BH) corrected *p*-value < 0.05] was applied to evaluate whether the alpha diversities of the urban gut microbiota were significantly different from those obtained in tribal gut microbiota.

### Ordination Analysis Using PCoA

PCoA analysis was performed on tribal and urban samples using the Jensen–Shannon (JS) divergence and partitioning-around-medoid (PAM) clustering methodology. Following this approach, the optimal number of clusters was estimated using the Calinski–Harabasz (CH) index (Arumugam et al., 2011). Additionally, PCoA analysis using weighted and un-weighted uniFrac distance was also performed using “phyloseq” package version 1.22.3 in R environment version 3.2.4 to check coherence in the obtained clustering pattern. Besides this, to elucidate the drivers for the clustering pattern, novel enterotypes were also identified among the two groups (urban and tribal) using “Dirichlet-Multinomial” package version 1.20.0 in R environment version 3.2.4 (Holmes et al., 2012).

### Inferring Functional Potential Contributed by Gut Bacterial Microbiota

Imputed functional profiles of the bacterial gut microbiota were inferred from 16S rRNA RDP abundance data at the genera level using the iVikodak platform (Nagpal et al., 2019). The abundances of KEGG pathways were obtained at all the KEGG hierarchy levels. All of the three modules, viz., Global Mapper, Local Mapper, and ISFA, provided by iVikodak were run on the taxonomic relative abundance data to get the functional prediction results required for downstream analysis.

### Core Feature Identification

Microbial taxa (OTUs/genera) that were consistently represented at a minimum abundance of 0.1% in at least 70% of the samples were affiliated as “core” taxa. In order to increase the confidence of the analysis, a bootstrapping approach was designed wherein a random set of samples were drawn from the whole set of samples, and a core set was collected from the randomly drawn sample set. This approach was iterated 1000 times to arrive at 1000 core sets for the whole sample set. A union of the core sets for all 1000 iterations was generated, and a bootstrap score was assigned to each feature based upon the frequency of appearance as a core feature in all iterations. The bootstrap score was scaled between 0 and 100, wherein a score of 100 for a feature indicated that the given taxon appeared as core in all 1000 iterations. In this study, only those taxa were considered as core that had a minimum bootstrap score of 80 in either the tribal or the urban cohort (Ganju et al., 2016). Information relating to core functions was procured from the iVikodak results.

### Differentiating Microbes and Functions

The relative abundance data of the taxa or functions abundant in at-least 51% samples, i.e., with non-zero median abundance in each cohort, were extracted and analyzed for identifying significantly different taxa/functions. A LDA approach, implemented within the LEfSe tool (Segata et al., 2011), was employed to identify significantly differentiating features (i.e., taxa/functions) between the two sample classes.

A RF classifier was also employed to construct a model in order to differentiate the gut microbiota composition in urban vs. tribal samples. Abundances of all the filtered bacterial genera/OTUs/functions identified in the urban and tribal microbiota samples were used as features when building their respective classifiers. The sample set (80 urban samples and 75 tribal samples) was randomly split into a training and a testing set in a proportion of 70:30, ensuring an equivalent proportion of urban and tribal samples in both the training and test sets. The training procedure involved 10-fold cross-validation with 10 replicates (i.e., 100 tests) (using R v.3.2.4, Random forest package v.4.6.14)^4^. The performances of the individual models were assessed with the “area under curve” (AUC) of the “receiver operating characteristics” (ROC) curve using the R pROC package^5^. The efficiencies of the model on the training and testing sets were plotted using the pROC package.

### Network Analysis

Correlation networks of filtered features (with non-zero median abundance) were generated for both urban and tribal cohorts from their respective abundance profiles, wherein initial correlation between each pair of microorganisms was calculated, and a pair of abundances of samples was swapped for each feature at one randomization step. Around 10000 randomization steps were executed, and the correlation between each pair of features was calculated at each randomization step. The expectancy value was calculated using the “expectancy-based method” in the tool network analysis for metagenomic abundance profiles (NAMAP) (Yadav et al., 2016). Positive and negative correlations between a pair of features were tagged according to the critical *r*-value obtained at the 99% confidence level. All correlation values (*r*) between the critical *r*-value and +1 were treated as positive correlations, and those between -1 and the negative of the critical *r*-value were treated as negative correlations. Values between the critical r-value and negative critical *r*-value were treated as insignificant correlations and did not contribute toward the creation of edges in the network. Cross-correlation networks were also constructed between diverse features such as differentiating genera, differentiating functions, and diversity to gain novel insights (Shankar et al., 2015). Correlations were plotted in Cytoscape 3.7.0 to visualize and study community network characteristics.

### Statistical Analysis

Non-parametric tests such as the Wilcoxon rank-sum test were used for checking the statistical significance between two groups. The false-discovery rate (FDR) was calculated using Benjamini-Hochberg for multiple test correction. For correlation analyses, the Spearman rank correlation test was used. Additionally, other statistical analyses and correlations between taxonomic or functional groups and qualitative or categorical metadata (with two groups or multiple groups) were performed using STAMP v2.1.3 (Parks et al., 2014). For multiple groups, the Kruskal-Wallis test was applied (Tukey-Kramer was used in for *post hoc* testing; the effect size was eta squared). Similarly, for two-group comparison, White’s non-parametric test was applied using STAMP.

## Data Availability Statement

Publicly available datasets were analyzed in this study. This data can be found here: https://www.nature.com/articles/srep18563#supplementary-information, PRJEB28572.

## Author Contributions

RS collated the data, performed the computational analysis, and interpreted the results with assistance from MH and SM. RS, MH, and SM prepared and reviewed the manuscript.

## Conflict of Interest

RS, MH, and SM are employed by Tata Consultancy Services.

## Footnotes

1 http://www.ncbi.nlm.nih.gov/Traces/sra/

2 https://cran.r-project.org/web/packages/RAM/

3 http://vegan.r-forge.r-project.org/

4 https://cran.r-project.org/web/packages/randomForest/randomForest.pdf

5 https://cran.r-project.org/web/packages/pROC/pROC.pdf

## Abbreviations

LPS: lipopolysaccharide
OTU: operational taxonomic unit
SCFA: short-chain fatty acid
TAD: tribal-associated diseases
TR: tribal
UAD: urban-associated diseases
UR: urban.

## References

[B1] Anand S. Kaur H. Mande S. S. (2016). Comparative *In silico* analysis of butyrate production pathways in gut commensals and pathogens. *Front. Microbiol.* 7:1945. 10.3389/fmicb.2016.01945 27994578 PMC5133246

[B2] Arokiasamy P. (2018). India’s escalating burden of non-communicable diseases. *Lancet Glob. Health* 6 e1262–e1263. 10.1016/s2214-109x(18)30448-0 30292427

[B3] Arumugam M. Raes J. Pelletier E. Le Paslier D. Yamada T. Mende D. R. (2011). Enterotypes of the human gut microbiome. *Nature* 473 174–180. 10.1038/nature09944 21508958 PMC3728647

[B4] Aßhauer K. P. Wemheuer B. Daniel R. Meinicke P. (2015). Tax4Fun: predicting functional profiles from metagenomic 16S rRNA data. *Bioinformatics* 31 2882–2884. 10.1093/bioinformatics/btv287 25957349 PMC4547618

[B5] Balamurugan R. Rajendiran E. George S. Samuel G. V. Ramakrishna B. S. (2008). Real-time polymerase chain reaction quantification of specific butyrate-producing bacteria, *Desulfovibrio* and *Enterococcus faecalis* in the feces of patients with colorectal cancer. *J. Gastroenterol. Hepatol.* 23(8 Pt 1), 1298–1303. 10.1111/j.1440-1746.2008.05490.x 18624900

[B6] Barone M. Turroni S. Rampelli S. Soverini M. D’Amico F. Biagi E. (2018). Gut microbiome response to a modern Paleolithic diet in a Western lifestyle context. *bioRxiv* [Preprint]. 10.1101/494187 31393934 PMC6687155

[B7] Bose T. Haque M. M. Reddy C. Mande S. S. (2015). COGNIZER: a framework for functional annotation of metagenomic datasets. *PLoS One* 10:e0142102. 10.1371/journal.pone.0142102 26561344 PMC4641738

[B8] Breiman L. (2001). Random forests. *Mach. Learn.* 45 5–32. 10.1023/A:1010933404324

[B9] Cani P. D. Amar J. Iglesias M. A. Poggi M. Knauf C. Bastelica D. (2007). Metabolic endotoxemia initiates obesity and insulin resistance. *Diabetes Metab. Res. Rev.* 56 1761–1772. 10.2337/db06-1491 17456850

[B10] Cho I. Blaser M. J. (2012). The human microbiome: at the interface of health and disease. *Nat. Rev. Genet.* 13 260–270. 10.1038/nrg3182 22411464 PMC3418802

[B11] Chung S. Lapoint K. Martinez K. Kennedy A. Boysen Sandberg M. McIntosh M. K. (2006). Preadipocytes mediate lipopolysaccharide-induced inflammation and insulin resistance in primary cultures of newly differentiated human adipocytes. *Endocrinology* 147 5340–5351. 10.1210/en.2006-0536 16873530

[B12] Clemente J. C. Pehrsson E. C. Blaser M. J. Sandhu K. Gao Z. Wang B. (2015). The microbiome of uncontacted Amerindians. *Sci. Adv.* 1:e1500183. 10.1126/sciadv.1500183 26229982 PMC4517851

[B13] Collier P. Venables A. J. (2017). Urbanization in developing economies: the assessment. *Oxford Rev. Econ. Policy* 33 355–372. 10.1093/oxrep/grx035

[B14] Conlon M. A. Bird A. R. (2014). The impact of diet and lifestyle on gut microbiota and human health. *Nutrients* 7 17–44. 10.3390/nu7010017 25545101 PMC4303825

[B15] Das B. Ghosh T. S. Kedia S. Rampal R. Saxena S. Bag S. (2018). Analysis of the gut microbiome of rural and urban healthy Indians living in sea level and high altitude areas. *Sci. Rep.* 8:10104. 10.1038/s41598-018-28550-3 29973712 PMC6031670

[B16] De Filippis F. Pasolli E. Tett A. Tarallo S. Naccarati A. De Angelis M. (2019). Distinct genetic and functional traits of human intestinal *Prevotella copri* strains are associated with different habitual diets. *Cell Host Microbe* 25 444–453.e3. 10.1016/j.chom.2019.01.004 30799264

[B17] De Filippo C. Di Paola M. Ramazzotti M. Albanese D. Pieraccini G. Banci E. (2017). Diet, environments, and gut microbiota. A preliminary investigation in children living in rural and urban Burkina Faso and Italy. *Front. Microbiol.* 8:1979 10.3389/fmicb.2017.01979 PMC5645538 29081768

[B18] De Palma G. Nadal I. Medina M. Donat E. Ribes-Koninckx C. Calabuig M. (2010). Intestinal dysbiosis and reduced immunoglobulin-coated bacteria associated with coeliac disease in children. *BMC Microbiol.* 10:63. 10.1186/1471-2180-10-63 20181275 PMC2843610

[B19] Dehingia M. Thangjam Devi K. Talukdar N. C. Talukdar R. Reddy N. Mande S. S. (2015). Gut bacterial diversity of the tribes of India and comparison with the worldwide data. *Sci. Rep.* 5:18563. 10.1038/srep18563 26689136 PMC4686986

[B20] Dhakan D. B. Maji A. Sharma A. K. Saxena R. Pulikkan J. Grace T. (2019). The unique composition of Indian gut microbiome, gene catalogue, and associated fecal metabolome deciphered using multi-omics approaches. *Gigascience* 8:giz004. 10.1093/gigascience/giz004 30698687 PMC6394208

[B21] Dominguez-Bello M. G. Godoy-Vitorino F. Knight R. Blaser M. J. (2019). Role of the microbiome in human development. *Gut* 68 1108–1114. 10.1136/gutjnl-2018-317503 30670574 PMC6580755

[B22] Dubey A. K. Uppadhyaya N. Nilawe P. Chauhan N. Kumar S. Gupta U. A. (2018). LogMPIE, pan-India profiling of the human gut microbiome using 16S rRNA sequencing. *Sci. Data* 5:180232. 10.1038/sdata.2018.232 30375992 PMC6207063

[B23] Esquivel-Elizondo S. Ilhan Z. E. Garcia-Peña E. I. Krajmalnik-Brown R. (2017). Insights into butyrate production in a controlled fermentation system via gene predictions. *mSystems* 2:e00051-17. 10.1128/mSystems.00051-17 28761933 PMC5516221

[B24] Fortenberry J. D. (2013). The uses of race and ethnicity in human microbiome research. *Trends Microbiol.* 21 165–166. 10.1016/j.tim.2013.01.001 23566633

[B25] Furet J. P. Kong L. C. Tap J. Poitou C. Basdevant A. Bouillot J. L. (2010). Differential adaptation of human gut microbiota to bariatric surgery-induced weight loss: links with metabolic and low-grade inflammation markers. *Diabetes Metab. Res. Rev.* 59 3049–3057. 10.2337/db10-0253 20876719 PMC2992765

[B26] Ganju P. Nagpal S. Mohammed M. H. Nishal Kumar P. Pandey R. Natarajan V. T. (2016). Microbial community profiling shows dysbiosis in the lesional skin of Vitiligo subjects. *Sci. Rep.* 6:18761. 10.1038/srep18761 26758568 PMC4725359

[B27] Gupta P. N. S. (1980). Food consumption and nutrition of regional tribes of India. *Ecol. Food Nutr.* 9 93–108. 10.1080/03670244.1980.9990587

[B28] Hersoug L.-G. Møller P. Loft S. (2016). Gut microbiota-derived lipopolysaccharide uptake and trafficking to adipose tissue: implications for inflammation and obesity. *Obes. Rev.* 17 297–312. 10.1111/obr.12370 26712364

[B29] Holmes I. Harris K. Quince C. (2012). Dirichlet multinomial mixtures: generative models for microbial metagenomics. *PLoS One* 7:e30126. 10.1371/journal.pone.0030126 22319561 PMC3272020

[B30] Hunter P. (2012). The changing hypothesis of the gut. *EMBO Rep.* 13 498–500. 10.1038/embor.2012.68 22584354 PMC3367252

[B31] Kamble S. Y. Patil S. R. Sawant P. S. Sangita S. Pawar S. G. Singh E. A. (2010). *Studies on Plants Used in Traditional Medicine by Bhilla Tribe of Maharashtra.* Available at: http://nopr.niscair.res.in/bitstream/123456789/9797/1/IJTK 9(3) 591-598.pdf (accessed April 3, 2019).

[B32] Kapoor A. K. Dhall M. (2016). Poverty, malnutrition and biological dynamics among tribes of India. *Health Sci. J.* 10 1–5. 10.1016/S0140-6736(16)00345-7

[B33] Langille M. G. I. Zaneveld J. Caporaso J. G. McDonald D. Knights D. Reyes J. A. (2013). Predictive functional profiling of microbial communities using 16S rRNA marker gene sequences. *Nat. Biotechnol.* 31 814–821. 10.1038/nbt.2676 23975157 PMC3819121

[B34] Larsen J. M. (2017). The immune response to *Prevotella* bacteria in chronic inflammatory disease. *Immunology* 151 363–374. 10.1111/imm.12760 28542929 PMC5506432

[B35] Lin H. V. Frassetto A. Kowalik E. J. Jr. Nawrocki A. R. Lu M. M. Kosinski J. R. (2012). Butyrate and propionate protect against diet-Induced obesity and regulate gut hormones via free fatty acid receptor 3-independent mechanisms. *PLoS One* 7:e35240. 10.1371/journal.pone.0035240 22506074 PMC3323649

[B36] Lopez R. P. Hynes H. P. (2006). Obesity, physical activity, and the urban environment: public health research needs. *Environ. Health* 5:25. 16981988 10.1186/1476-069X-5-25 PMC1586006

[B37] Louis P. Flint H. J. (2009). Diversity, metabolism and microbial ecology of butyrate-producing bacteria from the human large intestine. *FEMS Microbiol. Lett.* 294 1–8. 10.1111/j.1574-6968.2009.01514.x 19222573

[B38] Manco M. Putignani L. Bottazzo G. F. (2010). Gut microbiota, lipopolysaccharides, and innate immunity in the pathogenesis of obesity and cardiovascular risk. *Endocr. Rev.* 31 817–844. 10.1210/er.2009-0030 20592272

[B39] Marchesi J. R. Adams D. H. Fava F. Hermes G. D. A. Hirschfield G. M. Hold G. (2016). The gut microbiota and host health: a new clinical frontier. *Gut* 65 330–339. 10.1136/gutjnl-2015-309990 26338727 PMC4752653

[B40] McNally C. P. Eng A. Noecker C. Gagne-Maynard W. C. Borenstein E. (2018). BURRITO: an interactive multi-omic tool for visualizing taxa-function relationships in microbiome data. *Front. Microbiol.* 9:365. 10.3389/fmicb.2018.00365 29545787 PMC5837987

[B41] Moeller A. H. (2017). The shrinking human gut microbiome. *Curr. Opin. Microbiol.* 38 30–35. 10.1016/j.mib.2017.04.002 28458095

[B42] Nagpal S. Haque M. M. Mande S. S. (2016). Vikodak - a modular framework for inferring functional potential of microbial communities from 16S metagenomic datasets. *PLoS One* 11:e0148347. 10.1371/journal.pone.0148347 26848568 PMC4746064

[B43] Nagpal S. Haque M. M. Singh R. Mande S. S. (2019). iVikodak—a platform and standard workflow for inferring, analyzing, comparing, and visualizing the functional potential of microbial communities. *Front. Microbiol.* 9:3336. 10.3389/fmicb.2018.03336 30692979 PMC6339920

[B44] Neish A. S. (2009). Microbes in gastrointestinal health and disease. *Gastroenterology* 136 65–80. 10.1053/j.gastro.2008.10.080 19026645 PMC2892787

[B45] Newtonraj A. Murugan N. Singh Z. Chauhan R. C. Velavan A. Mani M. (2017). Factors associated with physical inactivity among adult urban population of Puducherry, India: a population based cross-sectional study. *J. Clin. Diagn. Res.* 11 LC15–LC17. 10.7860/JCDR/2017/24028.9853 28658812 PMC5483714

[B46] Obregon-Tito A. J. Tito R. Y. Metcalf J. Sankaranarayanan K. Clemente J. C. Ursell L. K. (2015). Subsistence strategies in traditional societies distinguish gut microbiomes. *Nat. Commun.* 6:6505. 10.1038/ncomms7505 25807110 PMC4386023

[B47] Ou J. Carbonero F. Zoetendal E. G. DeLany J. P. Wang M. Newton K. (2013). Diet, microbiota, and microbial metabolites in colon cancer risk in rural Africans and African Americans1234. *Am. J. Clin. Nutr.* 98 111–120. 10.3945/ajcn.112.056689 23719549 PMC3683814

[B48] Parks D. H. Tyson G. W. Hugenholtz P. Beiko R. G. (2014). STAMP: statistical analysis of taxonomic and functional profiles. *Bioinformatics* 30 3123–3124. 10.1093/bioinformatics/btu494 25061070 PMC4609014

[B49] Paul K. Singh J. (2017). Emerging trends and patterns of self-reported morbidity in India: evidence from three rounds of national sample survey. *J. Health Popul. Nutr.* 36:32. 10.1186/s41043-017-0109-x 28793930 PMC5550946

[B50] Pérez-Burillo S. Pastoriza S. Jiménez-Hernández N. D’Auria G. Francino M. P. Rufián-Henares J. A. (2018). Effect of food thermal processing on the composition of the gut microbiota. *J. Agric. Food Chem.* 66 11500–11509. 10.1021/acs.jafc.8b04077 30346155

[B51] Popkin B. M. (1999). Urbanization lifestyle changes and the nutrition transition. *World Dev.* 27 1905–1916. 10.1016/s0305-750x(99)00094-7

[B52] Pryde S. E. Duncan S. H. Hold G. L. Stewart C. S. Flint H. J. (2002). The microbiology of butyrate formation in the human colon. *FEMS Microbiol. Lett.* 217 133–139. 10.1111/j.1574-6968.2002.tb11467.x 12480096

[B53] Rajilic-Stojanovic M. Biagi E. Heilig H. G. Kajander K. Kekkonen R. A. Tims S. (2011). Global and deep molecular analysis of microbiota signatures in fecal samples from patients with irritable bowel syndrome. *Gastroenterology* 141 1792–1801. 10.1053/j.gastro.2011.07.043 21820992

[B54] Ramachandran A. Snehalatha C. Latha E. Manoharan M. Vijay V. (1999). Impacts of urbanisation on the lifestyle and on the prevalence of diabetes in native Asian Indian population. *Diabetes Res. Clin. Pract.* 44 207–213. 10.1016/s0168-8227(99)00024-8 10462144

[B55] Rognes T. Flouri T. Nichols B. Quince C. Mahé F. (2016). VSEARCH: a versatile open source tool for metagenomics. *PeerJ* 4:e2584. 10.7717/peerj.2584 27781170 PMC5075697

[B56] Saravanan V. S. Ayessa Idenal M. Saiyed S. Saxena D. Gerke S. (2016). Urbanization and human health in urban India: institutional analysis of water-borne diseases in Ahmedabad. *Health Policy Plan.* 31 1089–1099. 10.1093/heapol/czw039 27126201

[B57] Satija A. Hu F. B. Bowen L. Bharathi A. V. Vaz M. Prabhakaran D. (2015). Dietary patterns in India and their association with obesity and central obesity. *Public Health Nutr.* 18 3031–3041. 10.1017/S1368980015000312 25697609 PMC4831640

[B58] Schmieder R. Edwards R. (2011). Quality control and preprocessing of metagenomic datasets. *Bioinformatics* 27 863–864. 10.1093/bioinformatics/btr026 21278185 PMC3051327

[B59] Schnorr S. L. Candela M. Rampelli S. Centanni M. Consolandi C. Basaglia G. (2014). Gut microbiome of the Hadza hunter-gatherers. *Nat. Commun.* 5:3654. 10.1038/ncomms4654 24736369 PMC3996546

[B60] Segata N. (2015). Gut microbiome: westernization and the disappearance of intestinal diversity. *Curr. Biol.* 25 R611–R613. 10.1016/j.cub.2015.05.040 26196489

[B61] Segata N. Izard J. Waldron L. Gevers D. Miropolsky L. Garrett W. S. (2011). Metagenomic biomarker discovery and explanation. *Genome Biol.* 12:R60. 10.1186/gb-2011-12-6-r60 21702898 PMC3218848

[B62] Shankar V. Homer D. Rigsbee L. Khamis H. J. Michail S. Raymer M. (2015). The networks of human gut microbe–metabolite associations are different between health and irritable bowel syndrome. *ISME J.* 9 1899–1903. 10.1038/ismej.2014.258 25635640 PMC4511929

[B63] Shetty S. A. Marathe N. P. Shouche Y. S. (2013). Opportunities and challenges for gut microbiome studies in the Indian population. *Microbiome* 1:24. 10.1186/2049-2618-1-24 24451035 PMC3971629

[B64] Singh R. K. Chang H.-W. Yan D. Lee K. M. Ucmak D. Wong K. (2017). Influence of diet on the gut microbiome and implications for human health. *J. Transl. Med.* 15:73. 10.1186/s12967-017-1175-y 28388917 PMC5385025

[B65] Sokol H. Pigneur B. Watterlot L. Lakhdari O. Bermudez-Humaran L. G. Gratadoux J. J. (2008). *Faecalibacterium prausnitzii* is an anti-inflammatory commensal bacterium identified by gut microbiota analysis of Crohn disease patients. *Proc. Natl. Acad. Sci. U.S.A.* 105 16731–16736. 10.1073/pnas.0804812105 18936492 PMC2575488

[B66] Tandon D. Haque M. M. Gote M. Jain M. Bhaduri A. Dubey A. K. (2019). A prospective randomized, double-blind, placebo-controlled, dose-response relationship study to investigate efficacy of fructo-oligosaccharides (FOS) on human gut microflora. *Front. Microbiol.* 9:5473. 10.1038/s41598-019-41837-3 30940833 PMC6445088

[B67] Tandon D. Haque M. M. Saravanan R. Shaikh S. Sriram P. Dubey A. K. (2018). A snapshot of gut microbiota of an adult urban population from Western region of India. *PLoS One* 13:e0195643. 10.1371/journal.pone.0195643 29624599 PMC5889170

[B68] Ticinesi A. Lauretani F. Milani C. Nouvenne A. Tana C. Del Rio D. (2017). Aging gut microbiota at the cross-road between nutrition, physical frailty, and sarcopenia: is there a gut–muscle axis? *Nutrients* 9:E1303. 10.3390/nu9121303 29189738 PMC5748753

[B69] Tripathy J. P. Thakur J. S. Jeet G. Chawla S. Jain S. Prasad R. (2016). Urban rural differences in diet, physical activity and obesity in India: are we witnessing the great Indian equalisation? Results from a cross-sectional STEPS survey. *BMC Public Health* 16:816. 10.1186/s12889-016-3489-8 27538686 PMC4989330

[B70] UN DESA (2018). *Revision of World Urbanization Prospects | Multimedia Library.* New York, NY: United Nations Department of Economic and Social Affairs.

[B71] Uniyal S. K. Singh K. Jamwal P. Lal B. (2006). Traditional use of medicinal plants among the tribal communities of Chhota Bhangal, Western Himalaya. *J. Ethnobiol. Ethnomed.* 2:14. 16545146 10.1186/1746-4269-2-14 PMC1435742

[B72] Voreades N. Kozil A. Weir T. L. (2014). Diet and the development of the human intestinal microbiome. *Front. Microbiol.* 5:494. 10.3389/fmicb.2014.00494 25295033 PMC4170138

[B73] Wang Q. Garrity G. M. Tiedje J. M. Cole J. R. (2007). Naïve bayesian classifier for rapid assignment of rRNA sequences into the new bacterial taxonomy. *Appl. Environ. Microbiol.* 73 5261–5267. 10.1128/aem.00062-07 17586664 PMC1950982

[B74] WHO | Diet nutrition and the prevention of chronic diseases (2003). *Report of the Joint WHO/FAO Expert Consultation WHO.* Available at: https://www.who.int/dietphysicalactivity/publications/trs916/summary/en/ (accessed March 18, 2019).

[B75] Yadav D. Ghosh T. S. Mande S. S. (2016). Global investigation of composition and interaction networks in gut microbiomes of individuals belonging to diverse geographies and age-groups. *Gut Pathog.* 8:17. 10.1186/s13099-016-0099-z 27158266 PMC4858888

[B76] Yatsunenko T. Rey F. E. Manary M. J. Trehan I. Dominguez-Bello M. G. Contreras M. (2012). Human gut microbiome viewed across age and geography. *Nature* 486 222–227. 10.1038/nature11053 22699611 PMC3376388

[B77] Zeevi D. Korem T. Godneva A. Bar N. Kurilshikov A. Lotan-Pompan M. (2019). Structural variation in the gut microbiome associates with host health. *Nature* 1 43–48. 10.1038/s41586-019-1065-y 30918406

