# Supplementary material for: Lifestyle-Induced Microbial Gradients: An Indian Perspective
Source: Front Microbiol. 2019 Dec 17;10:2874. doi: 10.3389/fmicb.2019.02874 (PMC6928055; doi:10.3389/fmicb.2019.02874)
Supplement: Supplementary file 1 [file Data_Sheet_1.pdf]

Lifestyle induced microbial gradients: An Indian perspective

Supplementary Figures

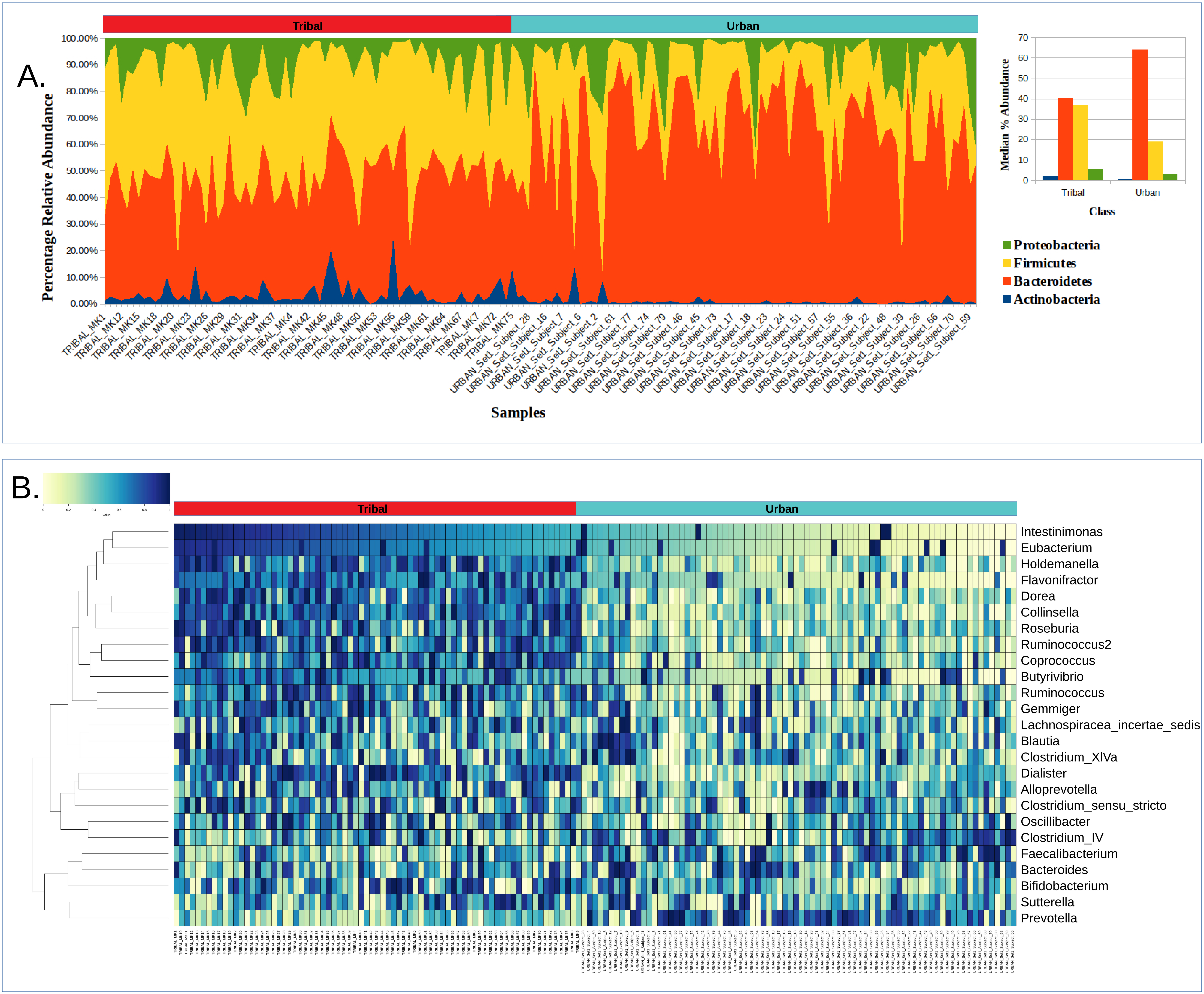

Supplementary Figure S1: Taxonomic distribution pattern of core phyla and genera

(A) Area plot representing relative abundance of core phyla observed in >70% of the samples across tribal and urban population under study. Bar plot shown as a subplot demonstrates change in median relative abundance of core phyla between urban and tribal groups (B) Heatmap demonstrates scaled relative abundance of core genera observed in >70% of the samples across tribal and urban population. *Prevotella* shows a stark difference while other distinct group of genera also formed clear clusters with tribal and urban group of samples

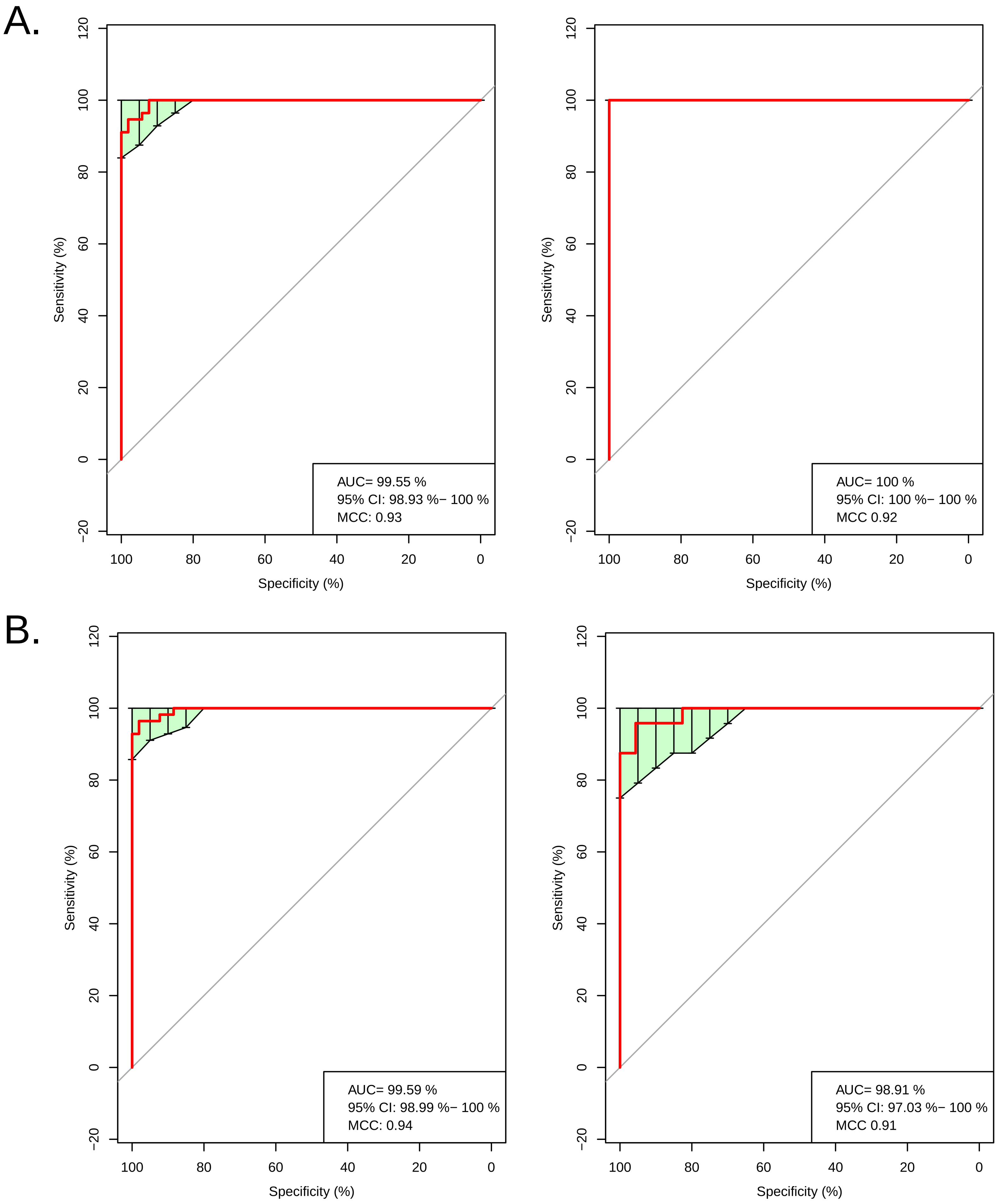

Supplementary Figure S2: Random forest classifier for differentiating between tribal and urban datasets.

Efficiency of classification was evaluated using Random Forest Classifier which computed AUC values of train and test data using (A) genus abundance data (B) OTU abundance data

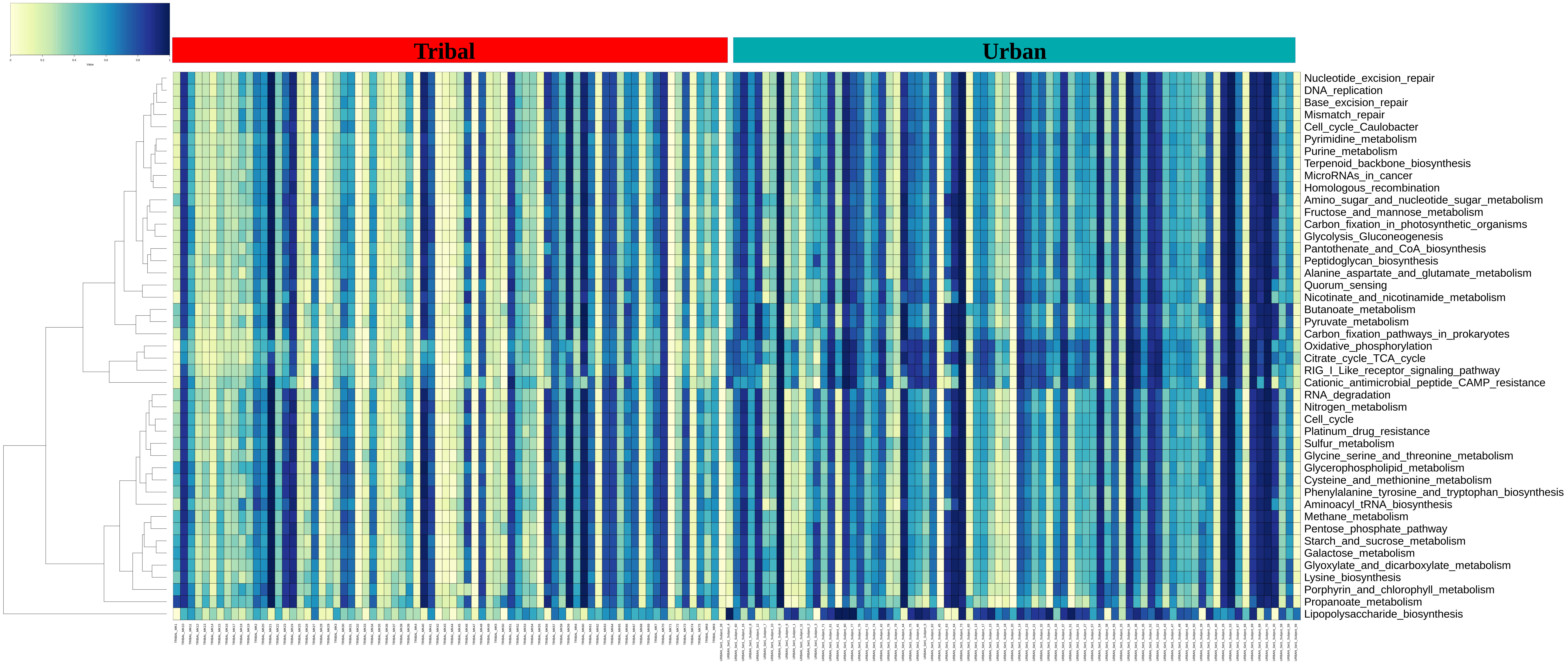

Supplementary Figure S3: Heat map representing distribution of rank-normalized abundances of the core functions across the two cohorts.
LPS biosynthesis pathway demonstrates consistently higher abundance in urban cohorts than in tribal counterparts along with other pathways such as TCA and OXPHOS

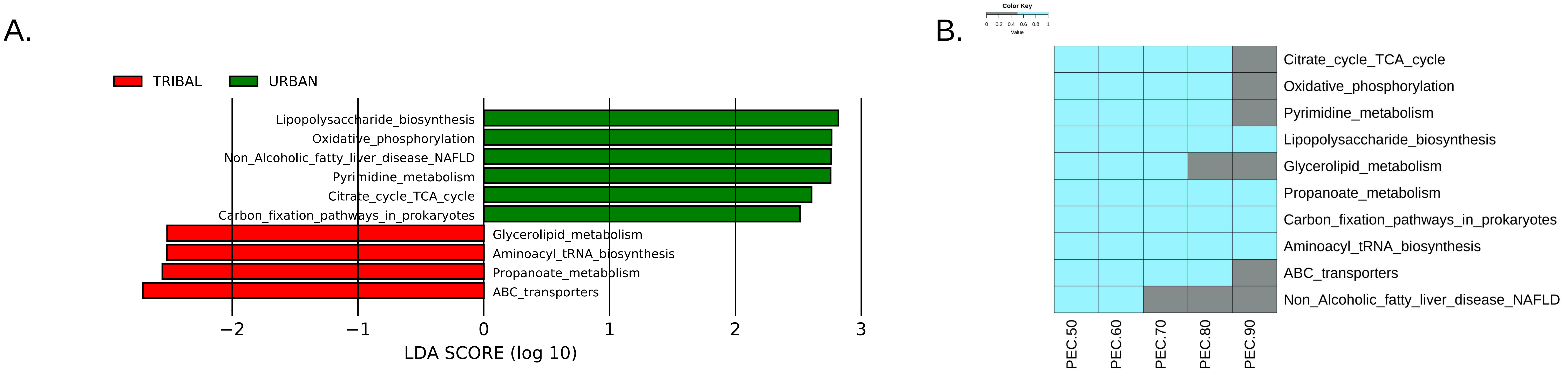

Supplementary Figure S4: Functions having a significantly different abundance (identified using LEfSe and iVikodak)
(A) Functions with significantly different abundance between tribal and urban gut microbiota identified using LEfSe with an LDA cut-off >2.5 at a p-value >0.001 (B) Pathway exclusion cut-off (PEC) were employed using iVikodak to assert a pathway to be significantly different only if it possesses> 80% enzymes involved in that particular pathway

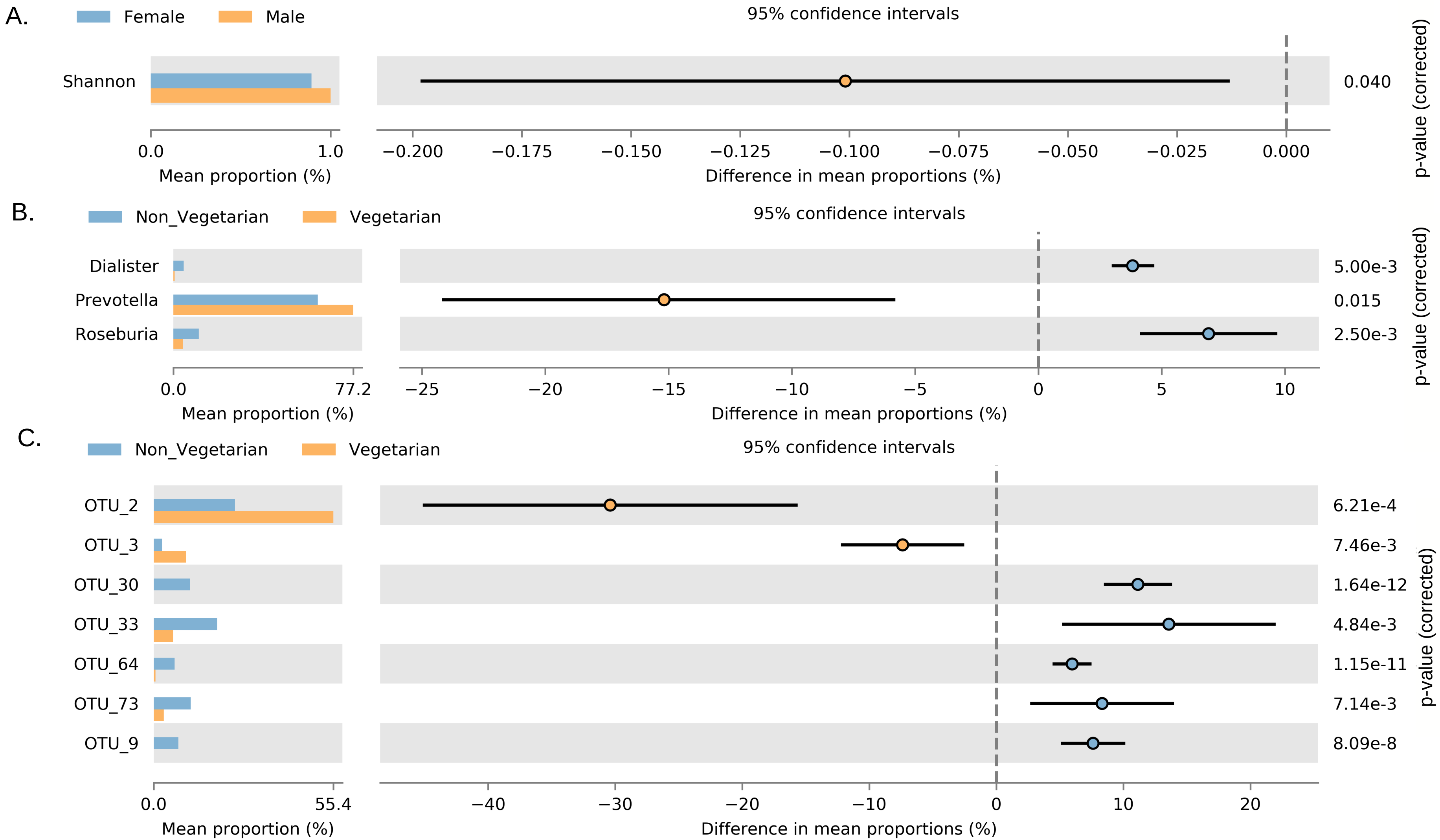

Supplementary Figure S5: **Diet and gender associated microbial variation**

Statistically significant associations of genera and differentiating OTUs with diet and gender were observed at FDR corrected p-value < 0.1 using STAMP (A) Shannon diversity was observed to be higher in males than in females. (B) Diet associated genera profiles indicates *Prevotella* to be higher in vegetarian individuals (C) Diet associated genera profiles

A.

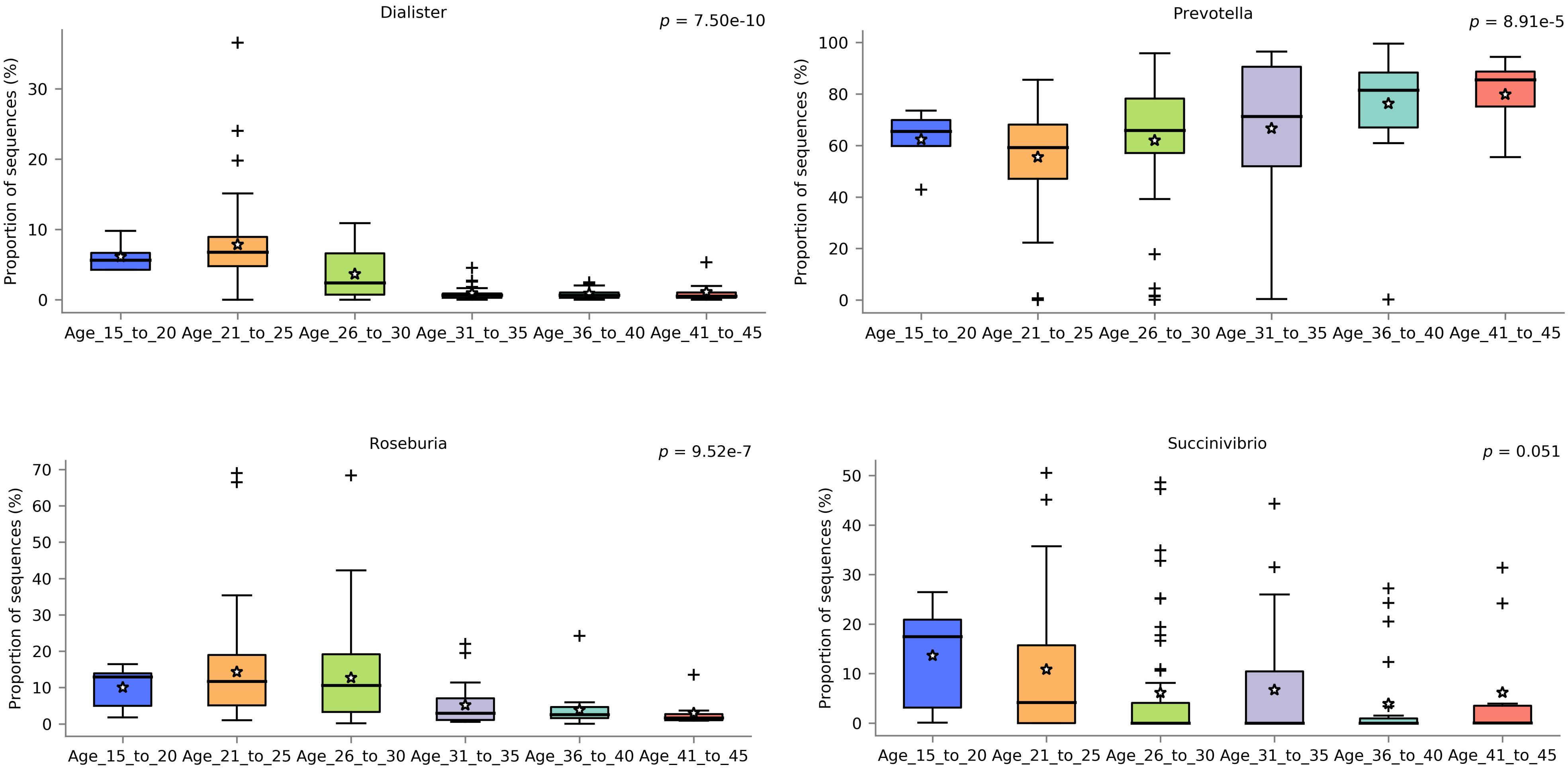

Dialister
p = 7.50e-10
Prevotella
p = 8.91e-5
Roseburia
p = 9.52e-7
Succinivibrio
p = 0.051
Proportion of sequences (%)
Age_15_to_20 Age_21_to_25 Age_26_to_30 Age_31_to_35 Age_36_to_40 Age_41_to_45

B.

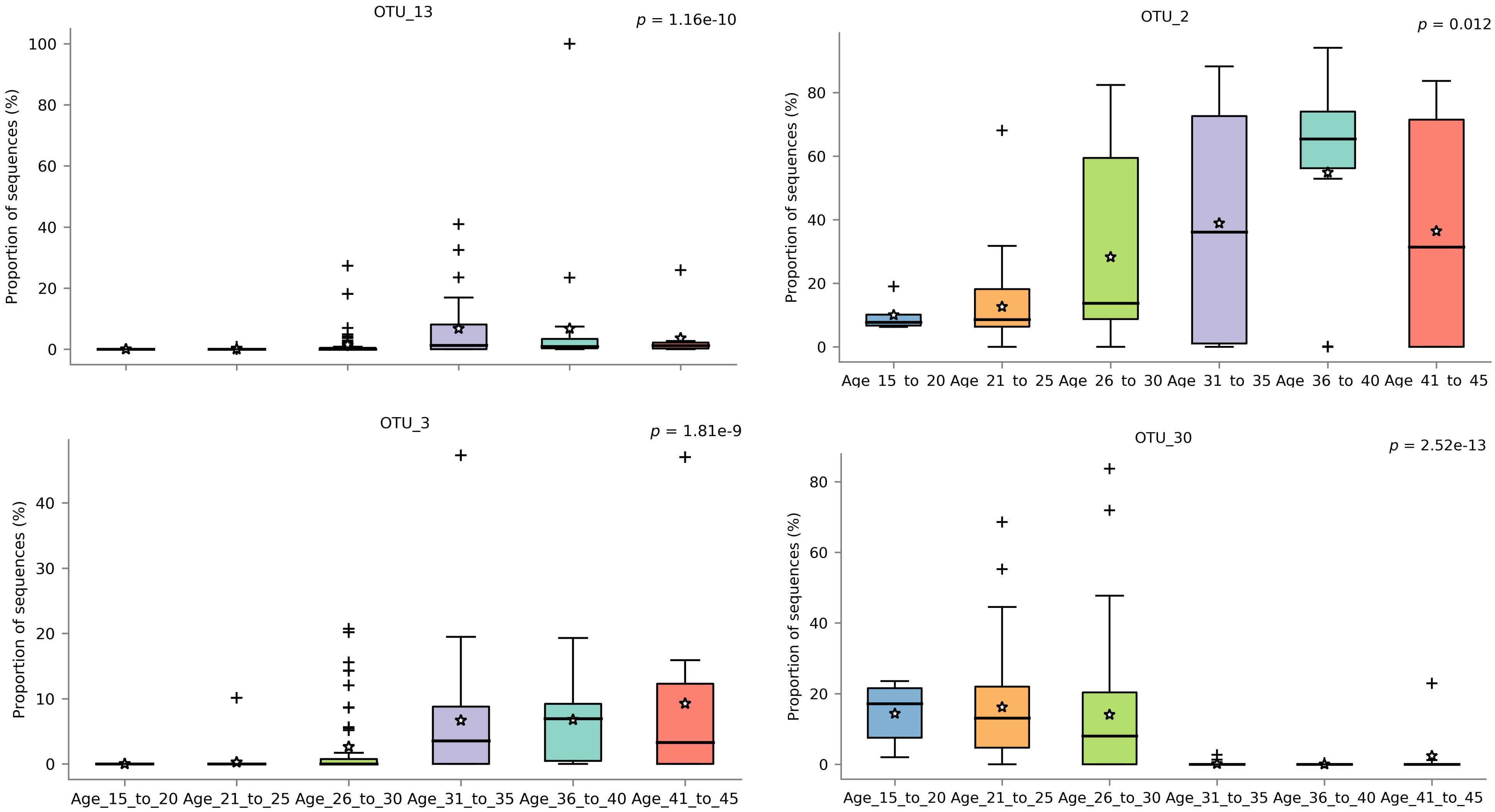
OTU_13
p = 1.16e-10
OTU_2
p = 0.012
OTU_3
p = 1.81e-9
OTU_30
p = 2.52e-13
Proportion of sequences (%)
Age_15_to_20
Age_21_to_25
Age_26_to_30
Age_31_to_35
Age_36_to_40
Age_41_to_45

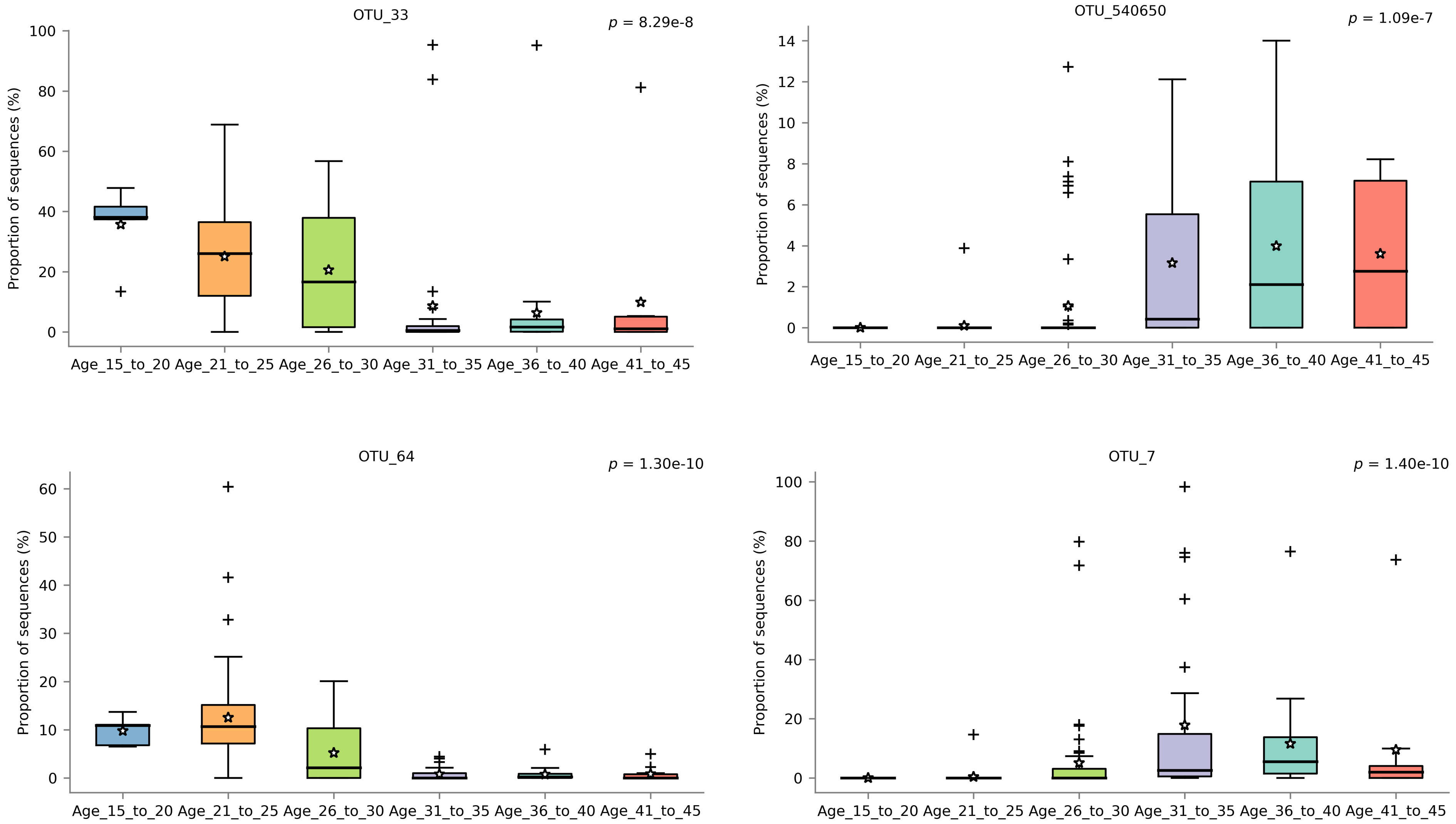

OTU_33
p = 8.29e-8
OTU_540650
p = 1.09e-7
OTU_64
p = 1.30e-10
OTU_7
p = 1.40e-10
Proportion of sequences (%)
Age_15_to_20
Age_21_to_25
Age_26_to_30
Age_31_to_35
Age_36_to_40
Age_41_to_45

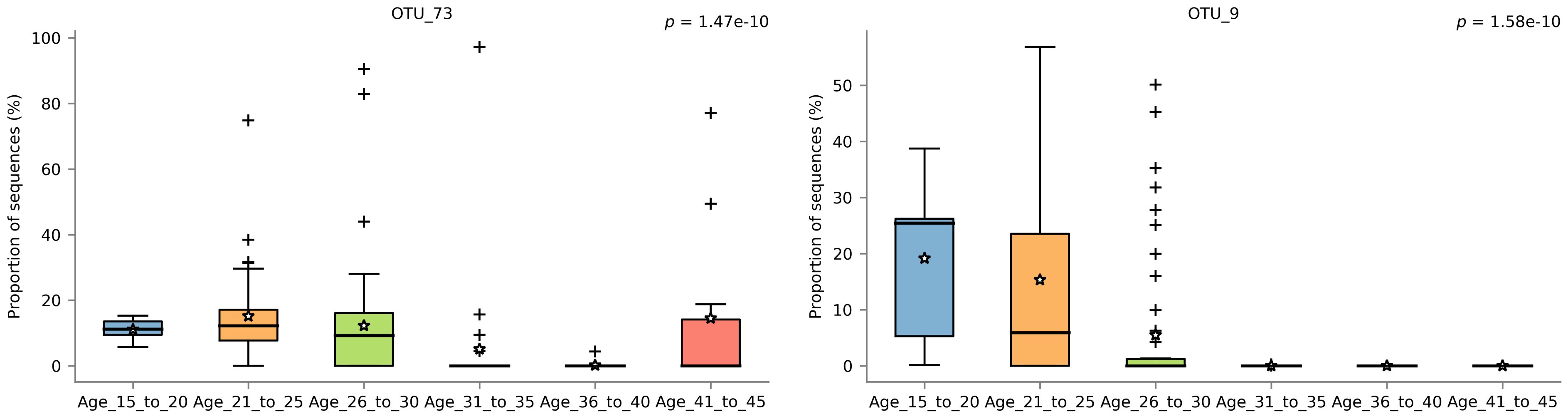

Supplementary Figure S6: Analysis of genera and OTU variations in different age groups

Statistically significant associations of genera and OTUs with different age groups were identified at FDR corrected p-value < 0.1 using STAMP (A) Box plots showing individual genera level variations with age (B) Box plots showing individual OTU level variations with age

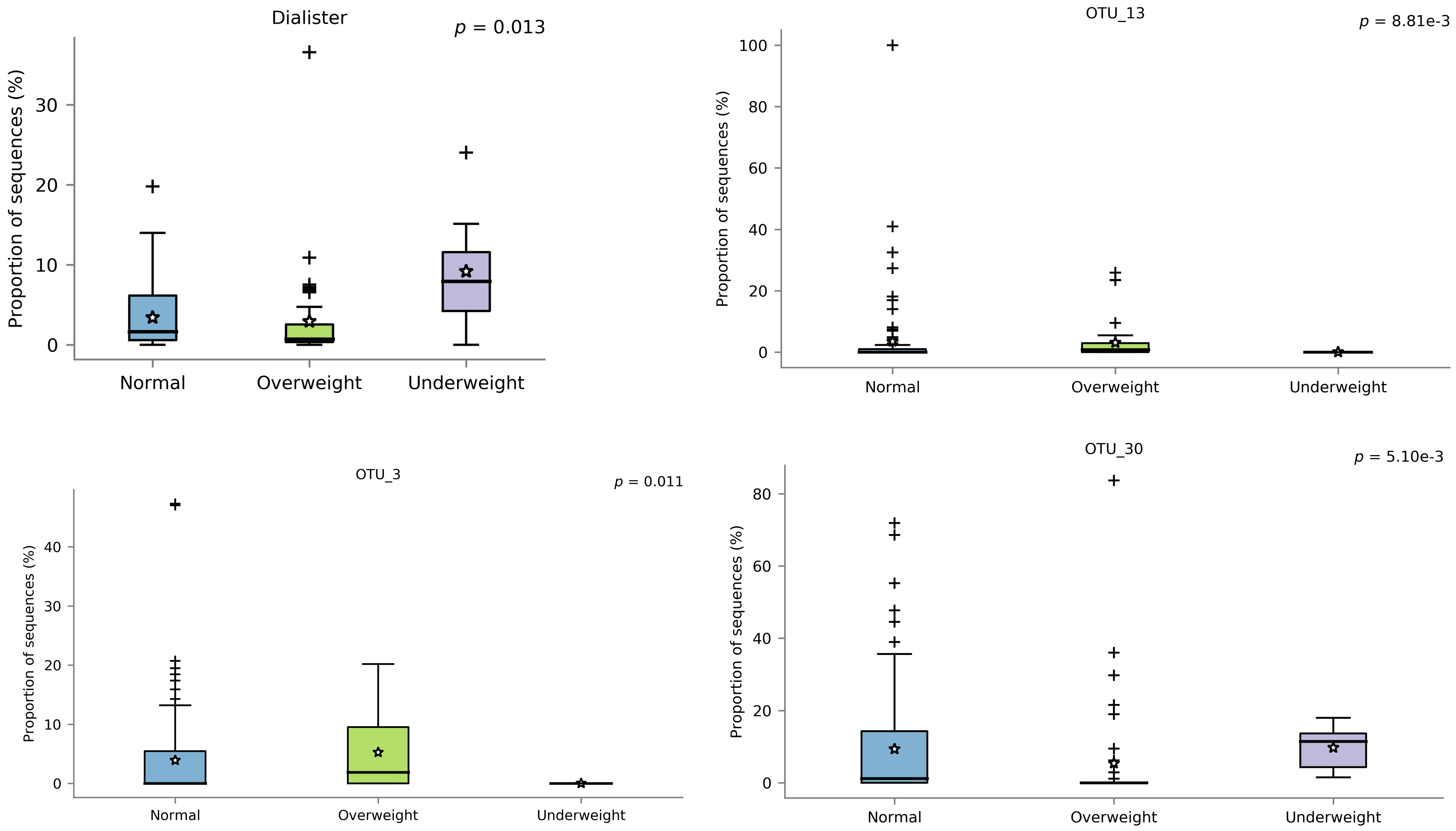
Dialister
p = 0.013
Proportion of sequences (%)
0
10
20
30
Normal
Overweight
Underweight
OTU_13
p = 8.81e-3
Proportion of sequences (%)
0
20
40
60
80
100
Normal
Overweight
Underweight
OTU_3
p = 0.011
Proportion of sequences (%)
0
10
20
30
40
Normal
Overweight
Underweight
OTU_30
p = 5.10e-3
Proportion of sequences (%)
0
20
40
60
80
Normal
Overweight
Underweight

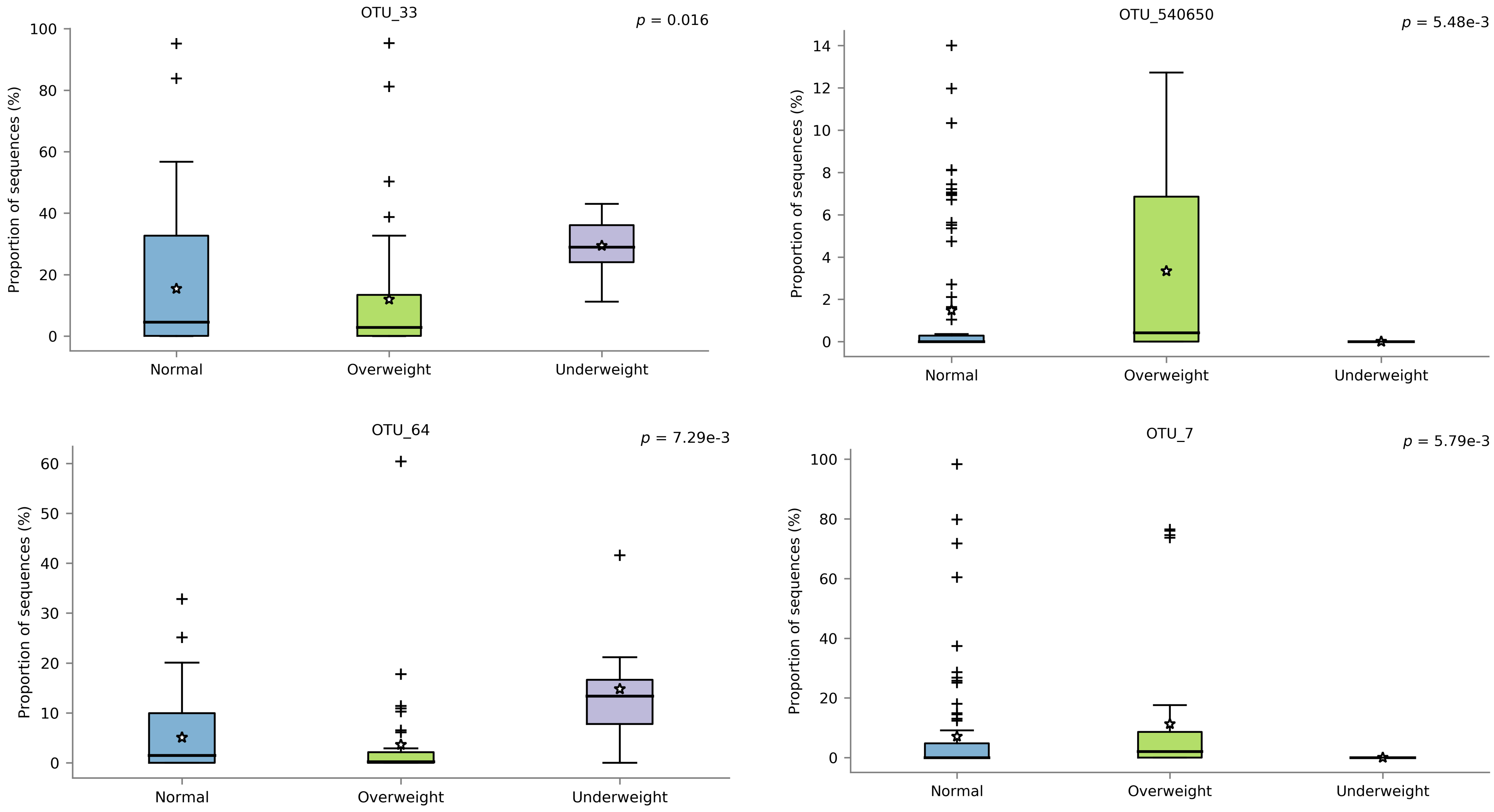
OTU_33
p = 0.016
OTU_540650
p = 5.48e-3
OTU_64
p = 7.29e-3
OTU_7
p = 5.79e-3
Proportion of sequences (%)
Normal
Overweight
Underweight
0
20
40
60
80
100
2
4
6
8
10
12
14
30
50

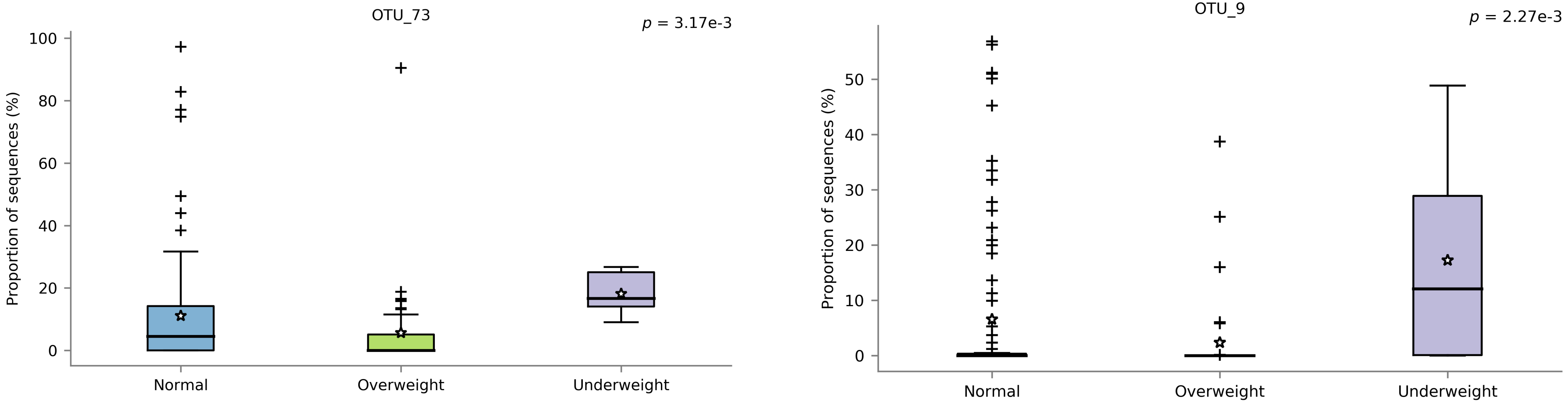

Supplementary Figure S7: Analysis of genera and OTU variations with BMI

Statistically significant associations of genera and OTUs with different BMI groups were observed at FDR corrected p-value < 0.1 using STAMP. Box plots showing individual genera and OTU level variations with BMI

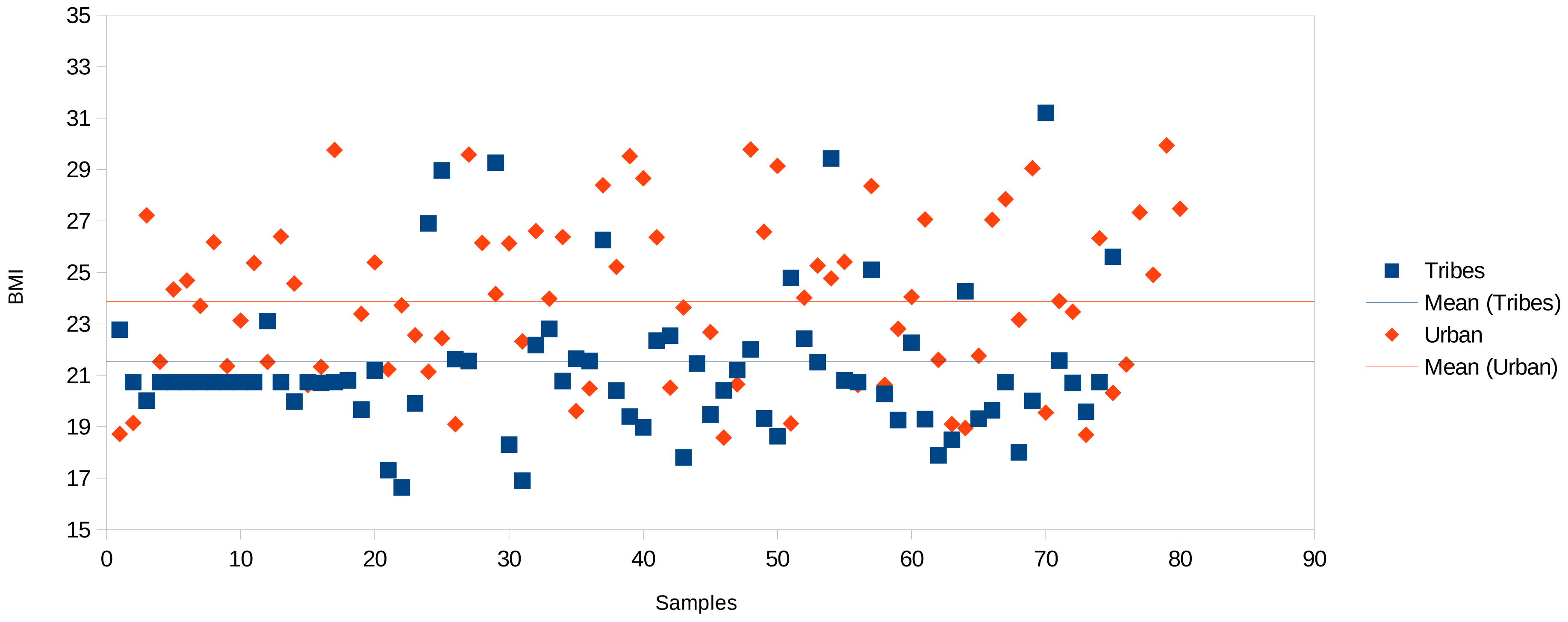

Supplementary Figure S8: Comparison of BMI values in tribal and urban cohorts

Variation in BMI values of samples pertaining to tribal and urban cohort. Overall urban population had higher mean BMI levels than tribal cohort
